# Essential Oils, *Pituranthos chloranthus* and *Teucrium ramosissimum*, Chemosensitize Resistant Human Uterine Sarcoma MES-SA/Dx5 Cells to Doxorubicin by Inducing Apoptosis and Targeting P-Glycoprotein

**DOI:** 10.3390/nu13051719

**Published:** 2021-05-19

**Authors:** Aida Lahmar, Aline Mathey, Virginie Aires, Dorra Elgueder, Anne Vejux, Rihab Khlifi, Fairouz Sioud, Leila Chekir-Ghedira, Dominique Delmas

**Affiliations:** 1 Research Unit Bioactive Natural Products and Biotechnology UR17ES49, Faculty of Dental Medicine of Monastir, University of Monastir, Avicenne Street, Monastir 5000, Tunisia; elahmar.aida@gmail.com (A.L.); elgueder.dorra@gmail.com (D.E.); rihabkhlifi@gmail.com (R.K.); fairouz.sioud@yahoo.fr (F.S.); l_chekir@yahoo.fr (L.C.-G.); 2 Université de Bourgogne Franche-Comté, F-21000 Dijon, France; aline.mathey52@gmail.com (A.M.); virginie.aires02@u-bourgogne.fr (V.A.); anne.vejux@u-bourgogne.fr (A.V.); 3 INSERM Research Center U1231—Cancer and Adaptive Immune Response Team, Bioactive Molecules and Health Research Group, F-21000 Dijon, France; 4 Biochemistry of the Peroxisome, Inflammation and Lipid Metabolism Team, EA 7270, F-21000 Dijon, France; 5 Centre Anticancéreux Georges François Leclerc, F-21000 Dijon, France

**Keywords:** doxorubicin, uterine sarcoma, P-glycoprotein, chemosensitization, essential oils

## Abstract

The multidrug resistance phenotype is a global phenomenon and causes chemotherapy failure in various cancers, such as in uterine sarcomas that have a high mortality rate. To overcome this phenotype, there is growing research interest in developing new treatment strategies. In this study, we highlight the potential of two essential oils from the Apiaceae family, *Pituranthos chloranthus* (PC) and *Teucrium ramosissimum* Desf. (TR), to act as chemopreventive and chemosensitizing agents against two uterine sarcoma cell lines, MES-SA and P-gp-overexpressing MES-SA/Dx5 cells. We found that PC and TR were able to inhibit the cell viability of sensitive MES-SA and resistant MES-SA/Dx5 cells by a slight modulation of the cell cycle and its regulators, but also through a significant induction of apoptosis. The molecular mechanism involved both caspase pathways associated with an overproduction of reactive oxygen species (ROS) and mitochondrial membrane depolarization. Very interestingly, the combination of doxorubicin with PC or TR induced a synergism to increase cell death in resistant MES-SA/Dx5 cells and, subsequently, had the benefit of decreasing the resistance index to doxorubicin. These synergistic effects were reinforced by a decrease in P-gp expression and its P-gp adenosine triphosphatase (ATPase) activity, which subsequently led to intracellular doxorubicin accumulation in resistant sarcoma cells.

## 1. Introduction

Historically, the majority of research on resistance to chemotherapy drugs has focused on intrinsic and acquired resistance mechanisms of tumor cells [1]. Intrinsic resistance is conditioned by the genetic heritage of cancer cells, resulting in ineffectiveness of initial chemotherapy, while acquired resistance is defined by chemotherapy-induced genetic changes [2]. Numerous studies conducted to date have focused on the analysis of multidrug resistance (MDR) induced in naive cells in the presence of cytotoxic molecules [3,4,5]. These molecular and cellular biology studies have shown that three major mechanisms are involved in drug resistance: (i) the potential reduction in the influx of water-soluble molecules through a transporter (e.g., 5-fluoro-uracil, methotrexate, cisplatin, doxorubicin), (ii) changes that affect the ability of cytotoxic drugs to kill cancer cells (e.g., alteration of the cell cycle, strengthening of DNA repair, inhibition of cell death, alteration of drug metabolism), and (iii) exacerbation of the active mechanisms of drug efflux [6,7]. Among the ATP-binding cassette (ABC) transporter superfamily, multidrug resistance proteins (MRPs), breast cancer resistance protein (BCRP/ATP Binding Cassette Subfamily G Member 2 (ABCG2)), and P-glycoprotein (P-gp/MDR1/ATP Binding Cassette Subfamily B Member 1 (ABCB1)), as ATP-driven efflux pumps, characterize classic MDR in cancer cells [3]. Thus, these efflux proteins are involved in the resistance to a wide variety of anticancer drugs (vinca-alkaloids, anthracyclines, taxanes, kinase inhibitors, etc.), with a possibility of cross-resistance to these drugs conferred by an overexpression of one ABC transporter [8]. This overexpression can result either from an inherent or acquired process: the former characterizes many cancers, especially colon cancer, which already significantly overexpresses the efflux transporters, and the latter involves a first chemotherapy inducing the overexpression of these carriers and resulting in ineffective chemotherapy [9,10].

In addition to colorectal cancer, the MDR phenotype also affects other cancers, such as uterine sarcoma, a rare and aggressive gynecologic malignancy worldwide with extremely short survival and poor prognosis [11]. Indeed, the combination of treatments used for this malignant neoplasm (surgery, radiotherapy, hormonal therapy, and chemotherapy) show a limited efficacy, mainly due to the early occurrence of metastasis and the onset of resistance mechanisms [12,13]. Moreover, concerning chemotherapy, various side effects often last a long time, such as cardiotoxicity, which can lead to cardiomyopathy and congestive heart failure, or acute myelotoxicity, thus limiting the use of commonly used anticancer agents such as doxorubicin (Adriamycin^®^, Dox) [14,15]. These therapeutic failures raise a triple challenge, which involves: (i) overcoming the resistance mechanisms induced by the use of chemotherapeutic drugs, and (ii) reducing the toxicity of these anticancer agents by lowering their concentrations, or (iii) increasing the chemosensitivity of malignant cells to these chemotherapeutic drugs.

In this context, we and others have previously shown that cytotoxic actions of various types of anticancer agents can be enhanced by the use of natural molecules such as polyphenols [16,17,18,19,20]. The molecular mechanisms involved in chemosensitization of cancer cells could include a modulation of xenobiotic-metabolizing enzymes as well as the induction of cell death pathways [9,21,22]. Among the potential compounds that could participate in this promising approach to counteract chemoresistance in human uterine sarcoma, two essential oils from the Apiaceae family growing in the dry and stony regions of southern Tunisia, *Pituranthos chloranthus* (PC) and *Teucrium ramosissimum* Desf. (TR), could be good candidates [23]. These essential oils were typically used for gastric ulcer and for the treatment of intestinal inflammation [24], and we have recently shown that PC and TR were able to enhance antibiotic actions toward clinical multidrug-resistant bacteria [23]. More specifically, some recent studies suggest the potential use of PC and TR as anticancer agents. Indeed, it has been shown that the major compound of TR extract, β-Eudesmol, inhibits proliferation, adhesion, and migration of human lung and colon cancer cell lines [25]. Concerning PC extract, few studies highlighted that one of the main bioactive compounds of this essential oil, terpinen-4-ol, exhibits growth inhibition on various malignancies and is able to potentialize drug efficacy by acting synergistically with several chemotherapeutics agents [26]. Furthermore, limonene, also one of the major components of PC extract, exerts therapeutic effects by inducing apoptosis on lung cancer cells and a xenograft animal model [27]. Altogether, these observations led us to explore the ability of these essential oils to counteract the chemoresistance of human uterine sarcoma to commonly used anticancer drugs such as Dox.

In the present study, we first investigated whether PC and TR were able to exert antiproliferative properties on two human uterine sarcoma cells lines, the sensitive MES-SA and the Dox-resistant variant MES-SA/Dx5 cell line, the model of choice for MDR modulator screening [28], in order to assess the potential use of these essential oils as chemopreventive and chemosensitizing agents. We next supposed that PC and TR are able to restore drug sensitization through the induction of the caspase-mediated apoptosis and oxidative stress, two mechanisms commonly activated by conventional chemotherapeutics drugs. We then evaluated whether the dose of Dox could be reduced when used in combination with PC and TR, while maintaining drug efficacy. Finally, we hypothesized that these essential oils chemosensitize MES-SA/Dx5 cells to Dox through the modulation of the overexpression and activity of the P-gp efflux transporter, allowing Dox to accumulate in MES-SA/Dx5 and exert in fine its antineoplastic activity on DNA.

## 2. Results

### 2.1. TR and PC Are Effective against the Resistance Index Conferred by MDR Human Uterine Sarcoma MES-SA/Dx5 Cells

**Figure 1:**
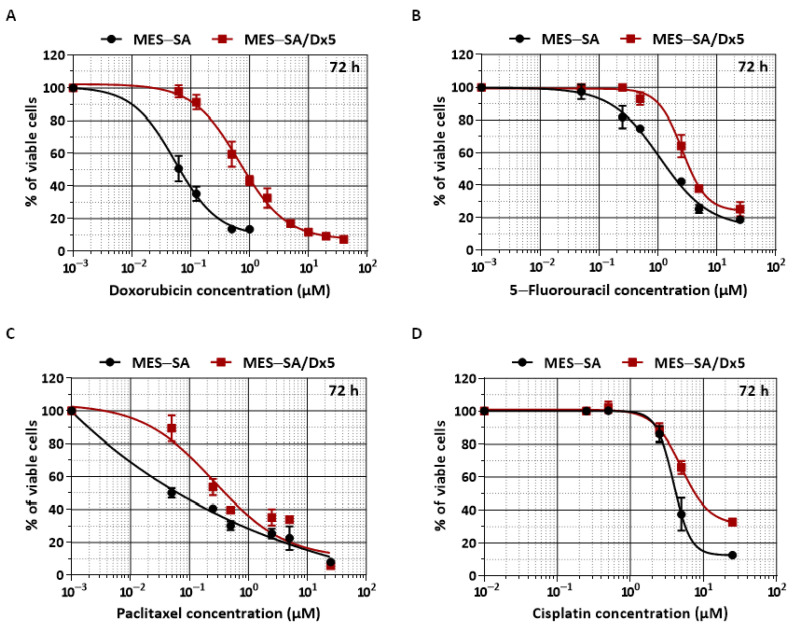
Differential sensitivity of MES-SA and MES-SA/Dx5 cells to various anticancer agents. Growth inhibition of MES-SA and MES-SA/Dx5 cells was determined using the (3-(4,5-dimethylthiazol-2-yl)-2,5-diphenyltetrazolium bromide (MTT) assay after 72 h of exposure to increasing concentrations of doxorubicin (**A**), 5-fluorouracil (**B**), paclitaxel (**C**), and cisplatin (**D**). The results are expressed as a mean percentage of control growth ± Standard Deviation (SD) of three independent experiments.

To determine whether essential oils from Apiaceae, PC, and TR (main constituents described in Supplementary Table S1) present potential chemopreventive or chemosensitivity properties against uterine sarcoma, we first evaluated their cytotoxic effects on two well-known human uterine sarcoma cell lines, MES-SA and MES-SA/Dx5. MES-SA is a poorly differentiated uterine sarcoma that was described initially as being sensitive to various chemotherapeutic agents such as Dox and paclitaxel [29]. MES-SA/Dx5 was obtained by long-term exposure of MES-SA cells to Dox [29,30] and overexpression of P-gp protein, conferring the MDR phenotype, which is reversed by stable sequence-specific MDR-1 gene silencing [31]. First of all, we evaluated the ability of MES-SA and its drug-resistant-derived MES-SA/Dx5 cell lines to present a differential cytotoxic response to Dox, as originally described by Harker and Sikic in 1985 [30]. After 48 and 72 h of treatment with various concentrations of Dox (from 0 to 40 µM), we observed that MES-SA/Dx5 presented a significant resistance to this drug (Supplementary Figure S1 and Figure 1A).

Indeed, the 50 percent inhibitory concentration of tumor cells (IC_50_) was 0.074 µM for MES-SA cells compared with 0.8 µM for MES-SA/Dx5 cells (Table 1). This confers a relative resistance to this drug by a factor of 10, as expressed by the resistance index (RI), which is the ratio of the IC_50_ concentrations between sensitive MES-SA and resistant MES-SA/Dx5 cells (Table 1). The overexpression of P-gp (ABCB1 or MDR-1) protein usually confers cross-resistance to various anticancer agents, and thus in the second step prior to the study of the cytotoxic effect of PC and TR, we tested the potential cross-resistance of MES-SA/Dx5 cells to other chemotherapeutic drugs. The use of three common anticancer drugs, 5-fluoro-uracil (5-FU), an antimetabolite agent, cisplatin, an alkylating agent, and paclitaxel, a taxane, did not present a significant difference between the uterine sarcoma cell lines in their IC_50_ after 72 h of treatment with a wide range of concentrations (Figure 1B–D). The relative resistance expressed by the IC_50_ ratio shows a very low or absent cross-resistance of MES-SA/Dx5 cells with other leading chemotherapeutic drugs. Indeed, cisplatin and 5-FU showed an RI of 1.21 and 2.38 respectively, after 72 h of treatment, whereas MES-SA/Dx5 cells show a moderated cross-resistance to paclitaxel with an RI of 3.8 (Table 1).

The absence of cross-resistance to certain classes of chemotherapies in the Dx5 variant may be explained by the differential mechanisms of action of the drugs and also perhaps by the subcellular localization or binding of drugs. This may also be due to differential affinities of drugs to P-gp and to their differential capacity to bind to the transporter, which may consequently result in various degrees of resistance.

**Figure 2:**
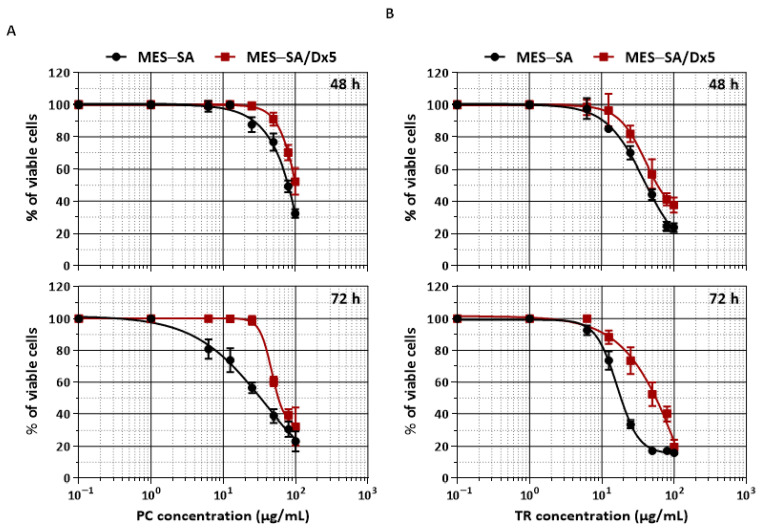
Inhibitory effects of *Pituranthos chloranthus* (PC) and *Teucrium ramosissimum* Desf. (TR) extracts on MES-SA and MES-SA/Dx5 cell proliferation. After treatment of MES-SA and MES-SA/Dx5 with increasing concentrations (0–100 μg/mL) for 48 and 72 h of PC (**A**) and TR (**B**), the percentage of cell viability was determined using the (3-(4,5-dimethylthiazol-2-yl)-2,5-diphenyltetrazolium bromide (MTT) assay. The results are expressed as a mean percentage of control growth ± Standard Deviation (SD) of three independent experiments.

To determine whether PC and TR have potential anticancer action, we then exposed MES-SA and MES-SA/Dx5 uterine sarcoma cells to increasing amounts of PC and TR (0–100 µg/mL) diluted in DMSO at 0.1% for 48 and 72 h, before determining cell viability by the MTT assay (Figure 2). Viability curves obtained after 48 and 72 h of treatment showed that PC and TR exert a concentration- and time-dependent cytotoxic activity in MES-SA cells, with greater efficacy for TR (Figure 2A,B).

The determination of the concentrations inhibiting 50% of cell viability (IC_50_) showed an IC_50_ for PC of 33.50 µg/mL, whereas TR showed an IC_50_ of 18.64 µg/mL at 72 h of treatment. Very interestingly, the same treatments carried out on resistant MES-SA/Dx5 cells showed a slight increase in the IC_50_ (Figure 2A,B, Table 2), but the RI was very low at 1.72 and 2.56 for PC and TR, respectively (Table 2).

**Figure 3:**
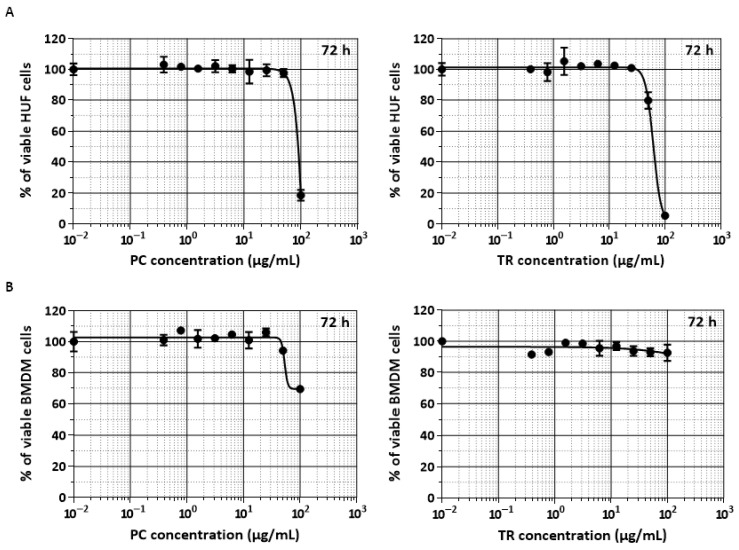
Effects of *Pituranthos chloranthus* (PC) and *Teucrium ramosissimum* Desf. (TR) extracts on normal primary human uterine fibroblast cells (HUF) and primary murine Bone Marrow-Derived Macrophages (BMDM) viability. After treatment of primary HUF and murine BMDM with increasing concentrations (0–100 µg/mL) of PC and TR for 72 h, the percentage of viable cells was assessed using the (3-(4,5-dimethylthiazol-2-yl)-2,5-diphenyltetrazolium bromide (MTT) assay. (**A**) Dose–response curves of PC-treated HUF (left panel) and TR-treated HUF (right panel). (**B**) Dose–response curves of PC-treated BMDM (left panel) and TR-treated BMDM (right panel). Data are expressed as a mean percentage of control growth ± Standard Deviation (SD) of two representative experiments (*n* = 6 replicates per concentration).

These first results indicated that there was no cross-resistance for PC and TR, with RI values comparable to those obtained for cisplatin and 5-FU (Table 1). The fact that MES-SA/Dx5 cells did not show cross-resistance toward PC and TR was of significant interest in the use of these extracts as chemopreventive or chemosensitizing agents. However, before statute on this potential chemosensitizing effect on tumor cells, it is relevant to compare the effects between tumoral and non-tumoral cells of the same type, which is therefore essential to define specific antitumor effects. In this way, we have exposed normal primary human uterine fibroblast cells (HUF) to increasing amounts of PC and TR (0–100 µg/mL) for 72 h as previously, before assessing cell viability (Figure 3A). We observed that neither PC nor TR presented a cytotoxic activity except at very high concentrations such as 100 µg/mL. The same was observed with another primary cell line of different type, that is primary murine Bone Marrow-Derived Macrophages (BMDM cells) (Figure 3B). In view of these results, we have chosen to work with extract concentrations, i.e., 12.5, 25, and 50 µg/mL, that do not induce significant toxicity in healthy cells but do have a sufficient effect in tumor cells, with 50 µg/mL being the highest concentration used in this study.

### 2.2. TR and PC Disturb the Cell Cycle and Its Key Regulators in MES-SA and MES-SA/Dx5 Cells

**Figure 4:**
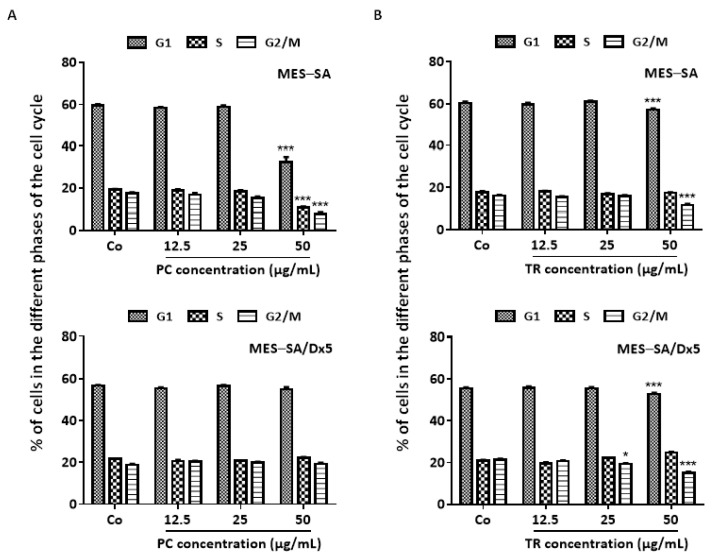
Quantifications of cell cycle distribution in MES-SA and MES-SA/Dx5 after a 72 h *Pituranthos chloranthus* (PC) and *Teucrium ramosissimum* Desf. (TR) extract treatment. Quantitative analysis of cell cycle distribution in PC-treated MES-SA and MES-SA/Dx5 (**A**) and TR-treated MES-SA and MES-SA/Dx5 (**B**). Data are expressed as mean ± Standard Error of the Mean (SEM) of three independent experiments; *p*-values were determined by a two-way ANOVA followed by Dunnett’s multiple comparison test. * *p* < 0.05 and *** *p* < 0.001 compared to control (Co) conditions. G1, Gap 1 phase; S, synthesis phase; G2/M, Gap 2/Mitosis phase.

To assess whether PC and TR-induced antiproliferative properties could be associated with a cell cycle arrest, we first analyzed the distribution of the two uterine sarcoma cells in the cell cycle using flow cytometry. MES-SA and MES-SA/Dx5 cells were exposed to two concentrations of PC and TR below the IC_50_ after 72 h of treatment (12.5 and 25 µg/mL) and one that is close to the IC_50_ (50 µg/mL). The results revealed that only the concentration close to the IC_50_ had a significant effect on the cell cycle of MES-SA cells with PC treatment (Figure 4A and Supplementary Figure S2A). We observed a marked decrease in the percentage of cells in the Gap 1 (G_1_), synthesis (S), and Gap 2/Mitosis (G_2_/M) phases. In addition, we observed a substantial subG_1_ peak reflecting the death of sarcoma MES-SA cells at 50 µg/mL of PC (Supplementary Figure S2A). By contrast, MES-SA/Dx5 cells did not display a modification of the cell distribution in the different cell cycle phases, which is comparable to the control whatever the concentration used after 72 h of treatment with PC (Figure 4A and Supplementary Figure S2A). Uterine cancer cells were also affected by a treatment with TR, showing a significant decrease in cells in the G_1_ and G_2_/M phases at the concentration close to the IC_50_ and a subsequent decrease in the cells in the G_1_ post-mitotic phase (Figure 4B and Supplementary Figure S2B). Very interestingly, contrary to PC, these TR effects at 50 µg/mL were also found on MES-SA/Dx5 cells, where the same phases were disturbed (Figure 4B).

**Figure 5:**
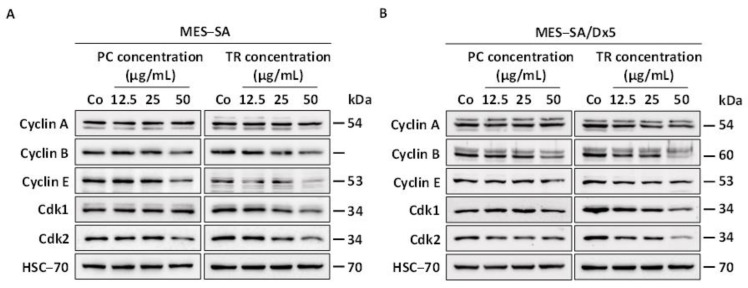
Modulation of key cell cycle regulators by *Pituranthos chloranthus* (PC) and *Teucrium ramosissimum* Desf. (TR) extracts in MES-SA and MES-SA/Dx5 cell lines. (**A**,**B**) Immunoblot analysis of cyclin A, B, E, Cyclin-dependent kinase 1 (Cdk1), and Cyclin-dependent kinase 2 (Cdk2) after a 72 h incubation period with vehicle (dimethyl sulfoxide, DMSO; Control, Co) or 12.5, 25, and 50 µg/mL of PC and TR in MES-SA (**A**) and MES-SA/Dx5 (**B**) cells. Heat shock cognate 70 (HSC-70) was used as the loading control. A representative blot from three independent experiments is shown.

The repartition of cells through the cell cycle is finely regulated by various key proteins, namely, cyclins and their partners, the cyclin-dependent kinases (Cdks) [32]. Therefore, we then explored whether the modulation observed in the distribution of cells by the two oil extracts was associated with disruption of the protein regulators involved in the progression of the cell cycle. Immunoblotting revealed that cyclin B and E protein expressions were decreased with 72 h of treatment with PC and TR in MES-SA cells (Figure 5A and Supplementary Figure S3A), consistent with a decrease in MES-SA cells in both the G_1_ and G_2_/M phases (Figure 4A,B). Interestingly, immunoblotting of Cdks (Cdk1 and Cdk2) controlling the main checkpoints of the cell cycle highlighted that their protein expressions were also modulated by treatment with Apiaceae extracts. Indeed, Cdk1, which is known to act in both the G_1_ and G_2_/M phases, was already reduced with TR treatment beginning at 25 µg/mL, which was accentuated with the increase in concentration at 50 µg/mL (Supplementary Figure S3A). Regarding the kinase Cdk2, its protein expression was reduced by both treatments with PC and TR, which was significant at 50 µg/mL for PC and starting at 25 µg/mL for TR (Supplementary Figure S3A). Similar to the MES-SA cell line, treatment with TR in the MES-SA/Dx5 cells led to a downward modulation of the protein expression of cyclins A, B, and E, as well as the protein expression of Cdk1 and 2 (Figure 5B and Supplementary Figure S3B).

Thus, these results are in agreement with those obtained in cell cycle experiments where TR also reduced the number of resistant cancer cells in phases G_1_ and G_2_/M (Figure 4B). On the other hand, it was very surprising to observe that PC and TR treatments on MES-SA/Dx5 cells resulted in a reproducible decrease in the protein expression of cyclins B and E and Cdk2 (Figure 5B and Supplementary Figure S3B). This modulation in the protein expression of cell cycle key regulators is probably too low to cause visible and significant repercussions on the distribution of MES-SA/Dx5 cells in the different phases of the cell cycle (Figure 4A,B).

### 2.3. TR and PC Induce Apoptosis through Caspase Activation in Both MES-SA and MES-SA/Dx5 Cells

**Figure 6:**
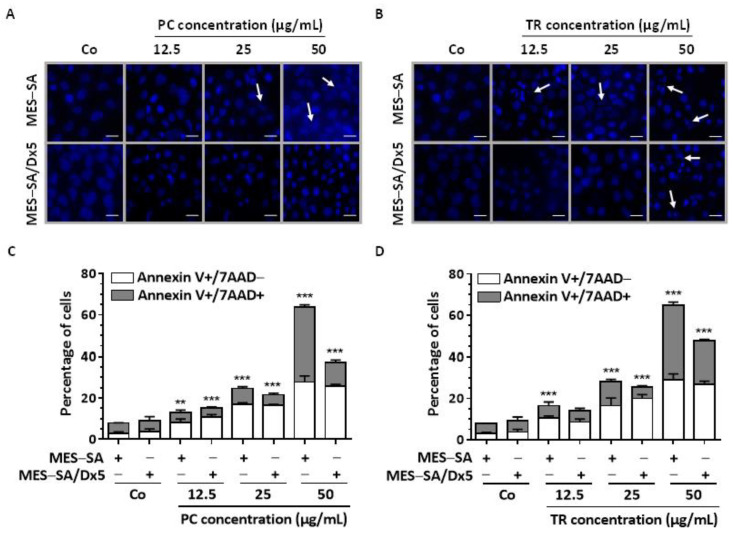
*Pituranthos chloranthus* (PC) and *Teucrium ramosissimum* Desf. (TR) extracts induce apoptosis of MES-SA and MES-SA/Dx5 cells. (**A**,**B**) Both uterine sarcoma MES-SA and MES-SA/Dx5 cell lines were stained with Hoechst 33342 after a 72 h treatment with vehicle (dimethyl sulfoxide, DMSO; Control, Co) or the indicated concentrations of PC (**A**) and TR (**B**). Representative fluorescence microscopy images of three independent experiments are shown (×40 magnification, scale bar = 10 µm). White arrows indicate typical condensed/fragmented apoptotic nuclei. (**C**,**D**) MES-SA and MES-SA/Dx5 cells were incubated for 72 h with medium containing vehicle (Co) or increasing concentrations of PC and TR, followed by annexin V/7-amino-actinomycin D (7AAD) staining. The percentages of early apoptotic cells (identified as the annexin V-positive/7AAD-negative population, white bar) and late apoptotic cells (identified as the annexin V-positive/7AAD-positive population, grey bar) were calculated after PC (**C**) and TR (**D**) treatment with the indicated concentrations for 72 h. Data are means ± Standard Deviation (SD) of three independent experiments; *p*-values were determined by a two-way ANOVA followed by Tukey’s multiple comparison test. ** *p* < 0.01, and *** *p* < 0.001 compared to control (Co) conditions of each cell line.

Our earlier study showed that many chemopreventive agents were able to slow down cell proliferation by modulating the cell cycle and/or by inducing a process of cell death via apoptosis [16,19,21,33]. These two molecular mechanisms can be involved independently of each other, thus contributing to the antitumor potential of the molecules [20]. We therefore investigated the ability of PC and TR to induce cell death in the MES-SA and MES-SA/Dx5 cell lines. Human uterine sarcoma cells stained with Hoechst 33342 demonstrated that both PC and TR induce typical morphological features of apoptosis, i.e., the condensation and fragmentation of the nuclear chromatin (as shown by the white arrows in Figure 6A,B). This process can be more accurately quantified using double staining with Annexin V/ 7-amino-actinomycin D (7-AAD) after treatment with PC and TR on the two cell lines (Figure 6C,D). This double staining allows the discrimination between early apoptotic cells (Annexin V +/7-AAD-) and late apoptotic cells that display double positivity for these dyes, since in advanced stages, cells can no longer exclude 7-AAD, which then bind to DNA with a high affinity. It appears that PC and TR treatments induce significant apoptosis in MES-SA cells beginning with 25 µg/mL, showing a greater increase at 50 µg/mL and reaching nearly 60% of apoptotic cells with a quasi-equal distribution between early and late apoptosis (Figure 6C,D). Very interestingly, both Apiaceae extracts were able to induce significant apoptosis in resistant MES-SA/Dx5 cells, reaching approximately 40% and 50% after PC and TR treatments with 50 µg/mL, respectively (Figure 6C,D). This last point is relevant because it could partly explain why the RI is very low in the resistant line treated with the two extracts. 

**Figure 7:**
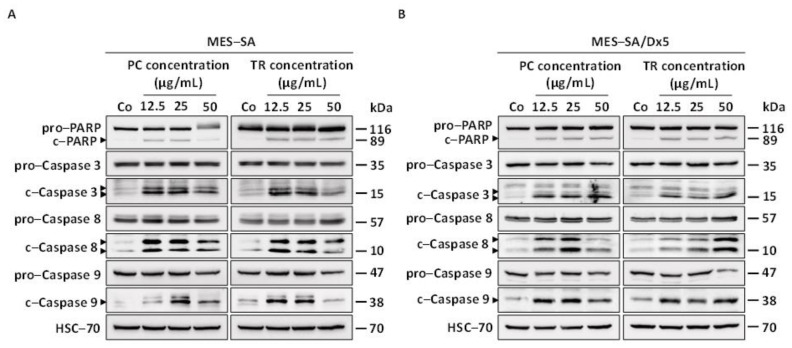
*Pituranthos chloranthus* (PC) and *Teucrium ramosissimum* Desf. (TR) extracts activate key players of the intrinsic apoptotic pathway in MES-SA and MES-SA/Dx5 cells. (**A**,**B**) Immunoblot analysis of pro and cleaved forms of Poly(ADP-ribose)-polymerase (PARP), caspases 3, 8, and 9 after 72 h of treatment with vehicle (dimethyl sulfoxide, DMSO; Control, Co) or the indicated concentrations of PC and TR in MES-SA (**A**) and MES-SA/Dx5 (**B**) cells. Heat shock cognate 70 (HSC-70) was used as the loading control. A representative blot from three independent experiments is shown.

We next explored the impact of PC and TR extracts on key molecular players of apoptotic pathways. Apiaceae-induced apoptosis was associated with Caspase 3 activation, as evidenced by the increased expression of its two cleaved fragments (17 and 19,000 relative molecular mass (*M_r_*)) and poly(ADP-ribose)polymerase (PARP) inactivation (increased expression of 89,000 *M_r_* cleaved fragment) in sensitive MES-SA cells after 72 h of treatment (Figure 7A and Supplementary Figure S4). Interestingly, PC and TR were able to activate all caspases involved in the extrinsic (Caspase 8) and intrinsic (Caspase 9) pathways. Indeed, immunoblotting showed that the 57,000 *M_r_* pro-form of Caspase 8 and the 47,000 *M_r_* pro-form of Caspase 9 were cleaved into their active fragments during the death process induced by the different concentrations of PC and TR (Figure 7A and Supplementary Figure S4). Apoptosis induction in MES-SA/Dx5 cells was also confirmed by immunoblotting where, in the same manner as in sensitive MES-SA cells, PC and TR induced activation of Caspase 8 and 9 and the activation of enzymes involved in the end of the caspase cascade, Caspase 3, and PARP (Figure 7B and Supplementary Figure S5).

### 2.4. TR and PC Modulate ROS Production and Mitochondrial Membrane Potential

**Figure 8:**
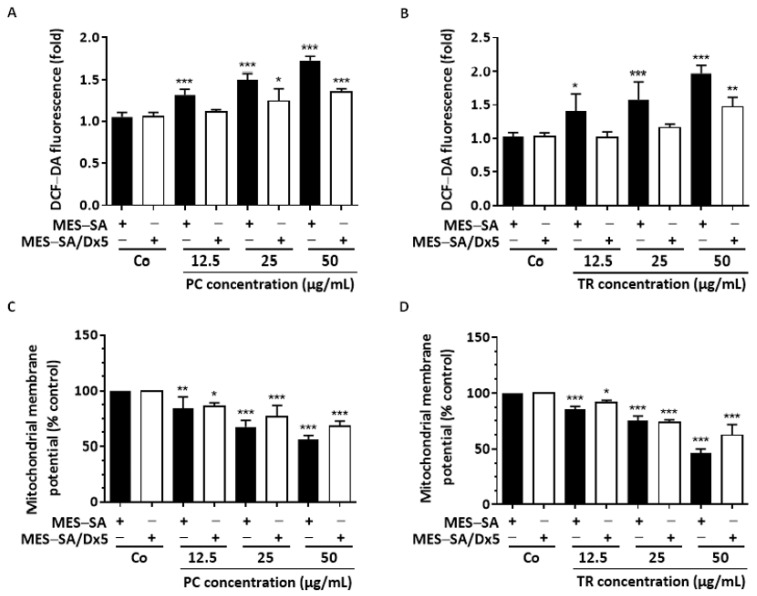
*Pituranthos chloranthus* (PC) and *Teucrium ramosissimum* Desf. (TR) extracts induce reactive oxygen species (ROS) accumulation and mitochondrial membrane potential (Δ_Ψ__m_) loss. (**A**,**B**) Both uterine sarcoma MES-SA and MES-SA/Dx5 cell lines were stained with DCFH after a 72 h treatment with vehicle (dimethyl sulfoxide, DMSO; Control, Co) or the indicated concentrations of PC (**A**) and TR (**B**). The fold changes of fluorescence intensity were relative to cells incubated with DCF with no drug treatment. (**C**,**D**) MES-SA and MES-SA/Dx5 cells were incubated for 72 h with medium containing vehicle (Co) or increasing concentrations of PC (**C**) and TR (**D**), followed by rhodamine 123 staining. Data are expressed as the mean ± Standard Deviation (SD) of three independent experiments; *p*-values were determined by a two-way ANOVA followed by Dunnett’s multiple comparison test. * *p* < 0.05, ** *p* < 0.01, and *** *p* < 0.001 compared to control (Co) conditions of each cell line.

Caspases are not the only major actors in triggering apoptosis: the redox state of cells usually plays a key role in various types of apoptotic cell deaths. Thus, excess of superoxide anion (O_2_^−^) and reactive oxygen species (ROS), which are key regulators in the oxidative stress process, were evaluated using 2’,7’-Dichlorodihydrofluorescein diacetate (H_2_DCF-DA) [34]. Cell-permeant H_2_DFC-DA is an indicator of intracellular ROS levels: This nonfluorescent probe can diffuse into the cell through the plasma membrane and is converted to a strongly emitting green fluorescent matter, dichlorofluorescein (DCF), upon intracellular oxidation [35]. We observed an increase in ROS levels with 12.5 µg/mL of PC and TR in MES-SA cells in a concentration-dependent manner (Figure 8A,B). These increases were strongly prevented in MES-SA/Dx5 cells, since only with a PC and TR concentration near IC_50_ was induction of a significant production of ROS possible, as compared with the control (Figure 8A,B). Very early changes at the cellular level can accompany this overproduction of ROS induced by various stimuli, including changes in the mitochondrial membrane potential (Δ_Ψm_) [19,33,34]. Inducers of apoptosis lead to a rapid loss of Δ_Ψm_ following permeability transition and disruption of the outer mitochondrial membrane [36]. In flow cytometry using the cationic fluorescent dye rhodamine 123, for which the rate of fluorescence decay is proportional to the mitochondrial membrane potential, we observed that PC and TR treatments induced, in a concentration-dependent manner, decreases in mitochondrial Δ_Ψm_ in MES-SA cells (Figure 8C,D). Although this decrease was lower in resistant MES-SA/Dx5 cells, it was still significant after 25 µg/mL of treatment (Figure 8C,D).

### 2.5. PC and TR Synergize with Doxorubicin to Reduce Viability Resistance of MES-SA/Dx5 Uterine Sarcoma Cells

**Figure 9:**
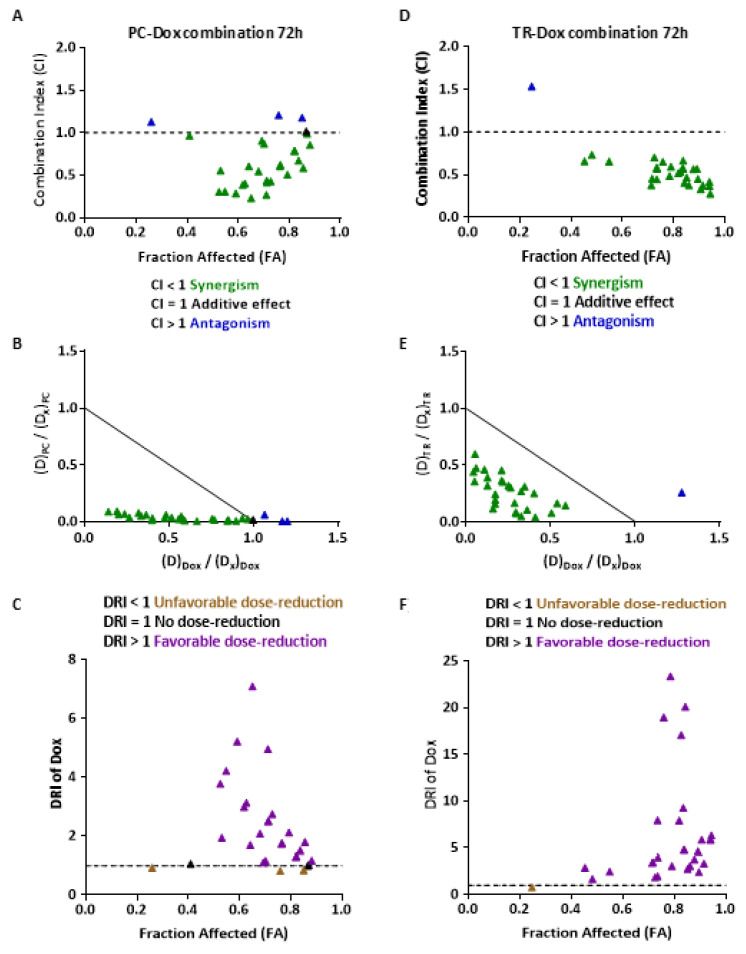
Determination of the combinatory effect of *Pituranthos chloranthus* (PC) and *Teucrium ramosissimum* Desf. (TR) extracts with doxorubicin (Dox) on MES-SA/Dx5 cell viability. Multidrug-resistant MES-SA/Dx5 cells were pretreated with increasing PC or TR concentrations for 24 h. Thereafter, the cells were treated with increasing Dox amounts for the last 48 h. Growth inhibition level of MES-SA/Dx5 cells was determined using the (3-(4,5-dimethylthiazol-2-yl)-2,5-diphenyltetrazolium bromide (MTT) assay. The cytotoxicity curves of single drugs and in combination were then simulated using CompuSyn software. (**A**) Fraction affected (FA) combination index (CI) plot for a 72 h PC–Dox combination. (**B**) Representative normalized isobologram plotted as the dose of each drug (D; PC and Dox) in combination inducing x% inhibition normalized with the dose at x% inhibition (D_x_) of single drugs on both the horizontal- and vertical-axes. (**C**) FA-dose reduction index (DRI) plot of Dox at different effect levels (for Dox/PC combinations). (**D**) Fraction affected (FA) combination index (CI) plot for a 72 h TR–Dox combination. (**E**) Representative normalized isobologram plotted as the dose of each drug (D; TR and Dox) in combination inducing x% inhibition normalized with the dose at x% inhibition (D_x_) of single drugs on both the horizontal- and vertical-axes. (**F**) FA-dose reduction index (DRI) plot of Dox at different effect levels (for Dox/TR combinations). DRI = 1 indicates no dose reduction, whereas DRI > 1 and <1 indicate respectively favorable and unfavorable dose reduction. The data of three independent experiments are shown.

To determine the ability of PC and TR to chemosensitize the MDR MES-SA/Dx5 cell line to Dox and the reverse Dox-resistant phenotype, we studied the potential synergistic effect of PC and TR with Dox in this cell line. MES-SA/Dx5 cells were pretreated for 24 h with increasing PC or TR concentrations and were then treated with increasing Dox concentrations for 24 and 48 h before the MTT cell proliferation assay. Concerning these chemosensitization studies, cells were not treated for more than 48 h with Dox in order to not induce too much cytotoxicity with this drug alone (Supplementary Figure S1). The equation of Chou–Talalay was used to calculate a combination index (CI), which reflects the interactions between PC and Dox (Supplementary Table S2) or TR (Supplementary Table S3) and Dox: CI values define synergism (CI < 1), additivity (CI = 1), and antagonism (CI > 1) between drugs [37,38]. The CI values for each combination point were then calculated and plotted against a growth inhibition potency, commonly named fraction affected (FA). Quantitative determination of drug interactions can also be analyzed by establishing a normalized isobologram plot in which each data point is defined as a CI value, where the several dose combinations of PC and Dox or TR and Dox were normalized with the dose of each single drug corresponding to a given x% growth inhibition. As illustrated in Supplementary Figure S6, the synergistic effects between PC and Dox were observed as early as 48 h, mostly drug combinations reducing cell viability from 50% to 80% (0.5 < FA < 0.8) (green dots; CI < 1; Supplementary Figure S6A). Moreover, the normalized isobologram revealed that the majority of combinations are synergistic (Supplementary Figure S6B). Concerning the 72 h incubation, most of the antagonistic combinations became synergistic and were associated with an increase in the mortality level (Figure 9A). Moreover, the corresponding normalized isobologram revealed that the synergistic PC–Dox combinations did not require high PC doses to achieve the same percentage of inhibition as that of PC used alone (Figure 9B). Since most anticancer drugs have a narrow therapeutic index, it is of particular interest to identify synergic drug combinations in which doses of each agent could be significantly decreased. These features can be estimated by a dose-reduction index (DRI), which is calculated from the inversion of the Chou–Talalay equation terms. Under PC exposure, as almost all DRI > 1, the dose of Dox can be reduced (Figure 9C). Concerning the 48 and 72 h TR–Dox combinations, similarly to PC–Dox combinations, most of the drug combinations showed synergistic effects, with a reduction in antagonistic effects and more synergistic effect levels after 72 h of incubation (Figure 9D and Supplementary Figure S6D). Furthermore, the dose of Dox in combination with TR can be strongly decreased (Figure 9F). Hence, using TR–Dox or PC–Dox combinations could potentially increase Dox therapeutic index, notably by reducing Dox side effects toward the host. Altogether, these results highlight that PC and TR can sensitize MES-SA/Dx5 cells to Dox.

### 2.6. TR Extracts Decreases P-gp Protein Expression and Its Activity, Resulting in Intracellular Dox Accumulation

**Figure 10:**
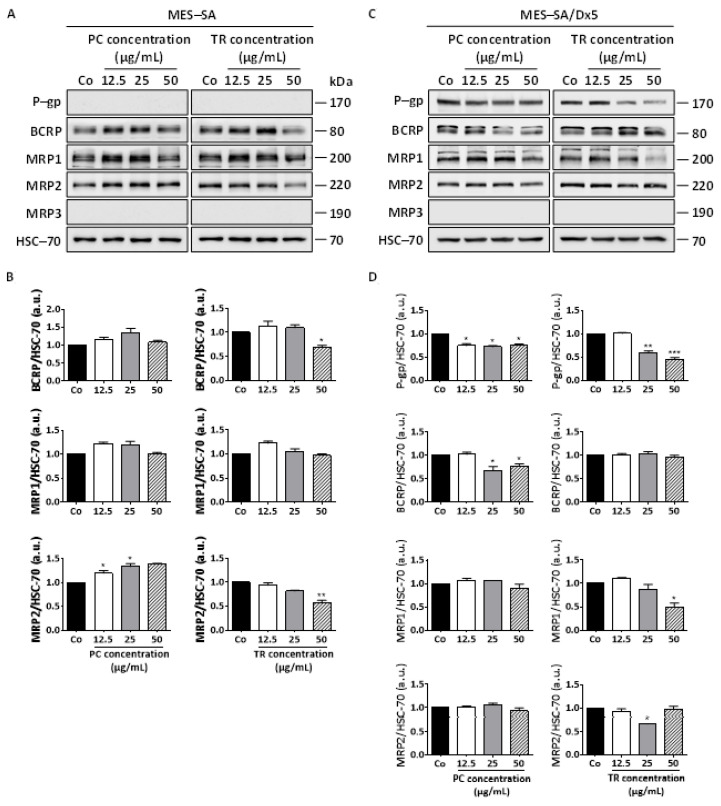
Differential expression of ABC transporters in MES-SA and MES-SA/Dx5 to *Pituranthos chloranthus* (PC) and *Teucrium ramosissimum* Desf. (TR) extracts. (**A**) Immunoblot analysis of P-glycoprotein (P-gp), breast cancer resistance protein (BCRP), and multidrug resistance proteins (MRP1, MRP2, and MRP3) after a 72 h treatment with vehicle (dimethyl sulfoxide, DMSO; Control, Co) or indicated concentrations of PC and TR in MES-SA cells. Heat shock cognate 70 (HSC-70) was used as the loading control. A representative blot from three independent experiments is shown. (**B**) Densitometric quantification of Western blotting obtained in (A). Data are expressed as mean fold induction ± Standard Error of the Mean (SEM) of three independent experiments; *p*-values were determined by a one-way ANOVA followed by Tukey’s multiple comparison test. * *p* < 0.05, ** *p* < 0.01, and *** *p* < 0.001 compared to control (Co) condition. F values of ANOVA tests (significant results): MRP2 expression, F = 15.53 and F = 28.46 for PC and TR, respectively; BCRP expression, F = 10.18 for TR. (**C**) Immunoblot analysis of P-gp, BCRP, MRP1, MRP2, and MRP3 after a 72 h treatment with vehicle (dimethyl sulfoxide, DMSO; Control, Co) or indicated concentrations of PC and TR in MES-SA/Dx5 cells. HSC-70 was used as the loading control. A representative blot from three independent experiments is shown. (**D**) Densitometric quantification of Western blotting obtained in (**C**). Data are expressed as mean fold induction ± Standard Error of the Mean (SEM) of three independent experiments; *p*-values were determined by a one-way ANOVA followed by Tukey’s multiple comparison test. * *p* < 0.05, ** *p* < 0.01, and *** *p* < 0.001 compared to control (Co) condition. F values of ANOVA tests (significant results): P-gp expression, F = 18.25 and 89.22 for PC and TR, respectively; BCRP expression, F = 9.96 for PC; MRP1 expression, F = 15.32 for TR; MRP2 expression, F = 16.57 for TR.

As we demonstrated previously, the ability of PC and TR extracts to sensitize resistant MES-SA/Dx5 cells could result from their ability to both induce a phenomenon of cell death in the same way as in sensitive MES-SA cells and to modulate ABC transporters, and more precisely the P-gp protein, which is overexpressed by the MES-SA/Dx5 cells, thus making them resistant to Dox. To explore this hypothesis, we first analyzed whether the base level of the main ABC proteins expressed in MES-SA and MES-SA/Dx5 cell lines is modulated by the Apiaceae extracts. As assumed, immunoblotting analysis revealed that there was no detectable P-gp protein expression in MES-SA cells (Figure 10A), whereas in contrast, MES-SA/Dx5 cells show a high expression of this ABC transporter (Figure 10C). Furthermore, among the main ABC transporters, MRP-3 (ABCC3) was not expressed in both cell lines, contrary to MRP-1 (ABCC1), MRP-2 (ABCC2), and BCRP (ABCG2), which were expressed in both cell lines (Figure 10A,C). Remarkably, only TR extract was able to decrease BCRP and MRP-2 protein expression significantly at 50 µg/mL in MES-SA cells (Figure 10A,B). However, the most striking and interesting fact to note is the capacity of the PC and TR extracts to significantly decrease the overexpression of P-gp in resistant MES-SA/Dx5 cells (Figure 10C), including from low concentrations of 12.5 µg/mL (Figure 10C,D), which would then be in agreement with the synergistic effects observed at low concentrations (Figure 9). As in MES-SA cells, TR extract was able to modulate MRP1 and MRP-2 protein expression (Figure 10D).

**Figure 11:**
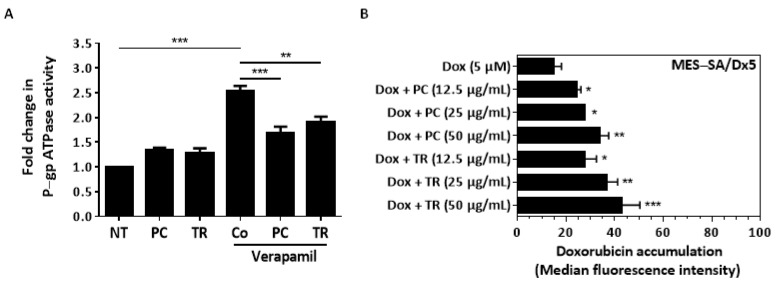
*Pituranthos chloranthus* (PC) and *Teucrium ramosissimum* Desf. (TR) extracts restore doxorubicin (Dox) sensitivity through a reduction in P-glycoprotein (P-gp)-mediated drug efflux. (**A**) Analysis of the effect of PC and TR alone or combined with verapamil on P-gp adenosine triphosphatase (ATPase) activity. Verapamil is used as a positive control by stimulating P-gp-mediated ATP hydrolysis. Since verapamil is a substrate for transport, PC and TR were tested to evaluate their capacity to reduce Pg-P activity by competing with verapamil. Drug-related P-gp ATPase activity is reported as a fold change relative to basal P-gp ATPase activity (untreated). The results are expressed as mean fold change ± Standard Error of the Mean (SEM) of one experiment performed in triplicate; *p*-values were determined by a one-way ANOVA followed by Tukey’s multiple comparison test. ** *p* < 0.01 and *** *p* < 0.001, with F value = 34.91. (**B**) Analysis of doxorubicin (Dox) uptake after a 1 h exposure of vehicle control (dimethyl sulfoxide, DMSO) or the PC and TR concentrations indicated, followed by incubation with 5 µM Dox at 37 °C for 6 h in MES-SA/Dx5 cells. Data are expressed as median of fluorescence intensity ± SEM of three independent experiments; *p*-values were determined by a one-way ANOVA followed by Tukey’s multiple comparison test. * *p* < 0.05, ** *p* < 0.01, and *** *p* < 0.001, with F value = 12.55.

This downward modulation of the overexpression of P-gp protein induced by the extracts must be related to its enzymatic activity and therefore the ability of this transporter to exclude the anticancer drug Dox. First, we evaluated the impact of PC and TR extracts on P-gp ATPase activity by measuring their capacity to decrease P-gp ATPase activity induced by a positive control substrate, verapamil. Using recombinant P-gp in a cell membrane fraction, we observed that verapamil induced a strong increase in P-gp ATPase activity, as a positive control. Indeed, PC and TR prevented the verapamil-stimulated ATPase activity induced by verapamil by 33.3% and 25.4%, respectively (Figure 11A). Together, the reduced P-gp ATPase activity and decreased protein expression of P-gp should allow for the accumulation of the anticancer drug Dox in cancer cells. When MES-SA/Dx5 cells were pretreated for 1 h with TR and PC before incubation with Dox for 6 h, we observed a significant increase of Dox in these cells in a concentration-dependent manner (Figure 11B). PC and TR could thus act as chemosensitizers to Dox through a disruption of P-gp activity.

## 3. Discussion

Although uterine sarcomas only account for 3.4% of all uterine corpus malignancies, they have a high mortality rate due to their very aggressive characteristics [39,40]. In spite of various therapeutic approaches (surgical treatment, chemotherapy, hormonotherapy, radiotherapy), the success rates in the management of uterine sarcomas remain low, in particular due to the emergence of treatment resistances and to a high risk of metastasis in advanced stages. Therefore, much effort has been made to date to improve the efficiency of existing treatments and/or to limit drugs’ side effects on non-cancerous cells, in order to improve cancer care and patient life quality. Among the major cause of failure in chemotherapy, multidrug resistance (MDR) phenotype plays a key role [41]. To overcome this tumor characteristic, research is focused on the development of new anti-cancer compounds, however, it comes up against the complexity of the mechanisms underlying MDR [42].

Indeed, various molecular mechanisms are involved in this MDR phenotype, particularly in uterine sarcoma, such as the loss of induction of apoptotic cell death pathways [43], the exacerbated phase I/II drug metabolism (i.e., increased expression and activity of metabolizing enzymes), or the deregulated expression and activity of plasma membrane phase III efflux transporters (ABC transporters). These events are often observed in clinic with the wide use of anticancer drugs such as doxorubicin (Dox), a first-line anticancer agent in sarcoma treatment [44]. However, its use is limited due to both the toxicity that Dox can induce and more particularly, cardiotoxicity (i.e., cardiomyopathy, congestive heart failure) and acute myelotoxicity [14,15], but also its lack of action on cancer cells following its efflux by the overexpression of carrier ABC, such as the P-gp protein in various cancer models [45,46,47]. Thus, a model of cells resistant to Dox by overexpression of P-gp protein was developed in uterine sarcoma, MES-SA/Dx5 cells [30], and for which, according to the literature, we have not observed cross-chemoresistance with other classical anticancer agents (Figure 1, Table 1). Thus, by using this model, some studies have attempted to evaluate the cellular response of new compounds or agents already used in therapeutics in order to counteract the resistance mechanism conferred by P-gp overexpression [48,49]. As mentioned above, the MDR phenotype can result from the dysfunction of several cellular and molecular mechanisms, so the search for new agents capable of acting on several of these mechanisms could be a valuable strategy to sensitize resistant cells and could offer opportunities for their potential use as therapeutic adjuvants. In this line, we evaluated the capacity of two essential oils extracts from south of Tunisia, *Pituranthos chloranthus* (PC) and *Teucrium ramosissimum Desf.* (TR), to act as a chemopreventive and chemosensitizing agents against the Dox-sensitive uterine sarcoma cell line MES-SA and the Dox-resistant variant cell line MES-SA/Dx5. We found that PC and TR were able to inhibit both cell viability of sensitive MES-SA and resistant MES-SA/Dx5 cells by a slight modulation of cell cycle and its regulators, but also by a significant induction of apoptosis. These mechanisms of cell viability inhibition through a disturbance of cell cycle progression and induction of apoptosis are part of the cytotoxic effects induced by naturally occurring molecules such as polyphenols (i.e., resveratrol and xanthohumol) [6,10,19]. The molecular mechanism involved the activation of intrinsic and extrinsic caspases associated with an overproduction of ROS by cancerous cells treated with PC or TR. This ROS production was accompanied by slight reproducible mitochondrial membrane depolarization, as shown by a rapid loss of Δ_Ψm_. The latter event is very interesting, since it is known that (i) overwhelming production of ROS disturbs Δ_Ψm_, inducing apoptotic-like cell death [50,51], and (ii) several studies have proved an indirect correlation between ROS generation and MDR transporter expression. An intracellular increase in ROS levels leads to a decreased intrinsic P-gp protein expression [52]. We observed that PC and TR treatments were able to increase ROS production and decrease P-gp protein expression, supporting a potential negative correlation between ROS levels and P-gp transporter expression. Furthermore, P-gp pumps extrude multiple compounds outside the cell, which thus limits intracellular drug concentration and favors MDR [53]. The most effective P-gp inhibitors in vitro, including verapamil and cyclosporine A, have shown serious side effects in clinical trials [54]. Here, we have demonstrated that PC and TR inhibited verapamil-stimulated P-gp ATPase activity and subsequently increased intracellular concentration of Dox in MES-SA/Dx5 cells. The two events could account for the chemosensitizing effects of PC and TR as a pretreatment. As demonstrated by the FA-CI plot and normalized isobolograms of the Dox–PC and Dox–TR combinations, we observed a predominantly synergistic effect (CI < 1) leading to an increase in cell death in resistant uterine sarcoma cells and the possibility to decrease Dox concentration while maintaining its efficacy, as revealed by the DRI curve of Dox. Besides this capacity to potentiate the action of doxorubicin and to reduce the dose of Dox (DRI) in uterine sarcoma cells overexpressing the MDR phenotype, it is interesting to note that these oils are relatively safe at the concentrations used, i.e., 12.5, 25, and 50 µg/mL, on normal uterine cells. Previously, other authors have shown that non-cancerous cell line HaCaT and primary spleen cells isolated from BALB/c mice presented a tolerance to the presence of essential oils in the medium at concentration below 20 µg/mL [23]. Moreover, we have also detected no cytotoxicity on primary macrophages after 72 h of treatment, whatever the concentration tested, except with the very high concentration of 100 µg/mL for PC extract. Furthermore, when experiments were performed in vivo in mice, we observed that PC could counteract some of the cisplatin-induced toxicity (acute kidney and liver injuries), supporting the notion that essential oils may exert a protective action in non-cancerous cells against some toxic effects of anticancer drugs [22]. Nevertheless, in order to support the notion that these oils, like many other natural compounds, present no or moderate cytotoxicity in non-malignant cells, and specifically target cancerous cells, investigations should be carried out in particular on normal uterine cells to further decipher molecular mechanisms underlying their specificity toward cancer cells. Recent works have been able to suggest the implication of membrane receptors such as integrins [55] that could be implicated in natural compounds’ endocytosis and triggering their signaling pathways [56], but this remains to be elucidated. However, to further support the potential beneficial antitumor effects of essential oils and their potential adjuvant properties, much attention should be paid to their pharmacokinetics in vivo in preclinical models or in humans. Indeed, the major concern with natural compounds is their low bioavailability and plasmatic half-life, which could limit their tissue distribution and effects. As we and others have previously described, natural compounds such as polyphenols, and particularly resveratrol, can undergo phase I/II functionalization and conjugation reactions, which give rise to the production of several glucurono- or sulfo-conjugates [16,57]. Major resveratrol metabolites were shown to accumulate in various target tissues, such as colon, where their accumulation could be compatible with tumor regression in patients after oral consumption, suggesting that even though subjected to intensive metabolism, metabolites could serve as parental molecule reservoirs sustaining biological activity in vivo [58,59]. Essential oils are a complex mixture of compounds comprising various terpenes and aromatic molecules, which complicates the evaluation of their pharmacokinetics and raises the question of which specific compound accounts for the biological effect or whether it is a result of synergism of all compounds present in the mixture, or even, do metabolites retain biological activity in vivo. Some data from literature reviewed in [60] have shown that limonene, a monocyclic monoterpene predominantly present in PC (Supplementary Table S1), presents low bioavailability and tissue distribution, but is mostly metabolized into perillic acid and perillyl alcohol, which have been shown to possess higher biological activity than the parental molecule, at least in in vitro studies and some rodent xenograft cancer models. Likewise, it was demonstrated that β-eudesmol, a major constituent of TR (Supplementary Table S1), has low plasmatic stability, bioavailability, and tissue distribution. Indeed, Plengsuriyakarn et al. have evaluated, in normal and Colangiocarcinoma (CCA)-xenografted nude mice, the pharmacokinetics of orally and intravenously delivered radiolabeled ^99m^Tc-β-eudesmol at the non-toxic dose of 100 mg/kg body weight [61]. Data show a short elimination half-life (T_1/2_) after intravenous injection (i.v.) and oral administration (o.a.), however, values were higher after o.a. of β-eudesmol in healthy mice (i.e., 21.16 min for i.v. and 276.26 min for o.a.). Interestingly, most of the pharmacokinetics parameters were lower in healthy compared to CCA-xenografted nude mice. After o.a. of ^99m^Tc-β-eudesmol (100 mg/kg body weight), authors showed that it accumulated mostly in the stomach (maximum accumulation after 30 min), the small intestine and the lungs (maximum accumulation after 2 h), large intestine (maximum accumulation after 4 h), and to a lesser extent, in the heart, liver, kidney, spleen, pancreas, and brain, in both healthy and CCA-xenografted nude mice. Of note, in CCA-xenografted nude mice, 8% of the injected dose per gram of organ was found after 2 h of o.a. in tumor, and a 30-day daily administration of β-eudesmol (100 mg/kg body weight) significantly reduced tumor growth and lung metastasis and prolonged mice survival by 64.4% compared to the control group. Hence, despite rapid systemic clearance, β-eudesmol displayed antitumor activity, but whether these effects were directly attributed to the compound itself or its metabolites was an issue not addressed by the authors.

Thus, the step forward before considering the clinical use of PC and TR in uterine carcinoma prevention and as a therapeutic adjuvant would be to evaluate their pharmacokinetics in vivo to notably determine whether uterine tissue could be targeted by these essential oils, and to study their metabolism in regard to their biological activity and safety.

## 4. Materials and Methods

### 4.1. Cell Lines and Cell Culture

Human uterine sarcoma cell lines MES-SA and MES-SA/Dx5 were purchased from the American Type Culture Collection (ATCC, Molsheim, France). The doxorubicin (Dox)-resistant variant cell line MES-SA/Dx5 was generated by continuous exposure of MES-SA cells to increasing Dox concentrations [30]. Cells were cultured in Roswell Park Memorial Institute (RPMI) 1640 medium containing L-glutamine supplemented with 10% fetal bovine serum (FBS) (Dutscher, Brumath, France). Primary human uterine fibroblast normal cells (HUF) were obtained from the ATCC. HUF cells were cultured in Fibroblast Basal Medium supplemented with Fibroblast Growth Kit-Low Serum (ATCC, Molsheim, France). Murine bone marrow-derived macrophages (BMDM), generated from C57BL/6J mice, were kindly provided by David Masson’s team (Institut National de la Santé et de la Recherche Médicale (INSERM) U1231, Dijon, France). Murine BMDM were cultured in RPMI 1640 medium containing L-glutamine supplemented with 10% FBS, 20% L929-conditioned medium, and antibiotics (100 U/mL penicillin, 100 µg/mL streptomycin) (Dutscher, Brumath, France). Cells were kept in a 5% CO_2_ humidified atmosphere at 37 °C. Mycoplasma potential contamination was assessed weekly by using the MycoAlert Mycoplasma Detection Kit (Lonza, Levallois-Perret, France). For the whole experiments, cells were seeded at a density of 14,000 cells/cm^2^ for MES-SA and MES-SA/Dx5 cells. BMDM and HUF cells were seeded respectively at a density of 125,000 cells/cm^2^ and 18,700 cells/cm^2^. Cells were allowed to recover for 24 h before treatments.

### 4.2. Chemical Reagents and Antibodies

Dox, cisplatin, 5-fluorouracil, and paclitaxel were purchased from Sigma-Aldrich (St. Quentin Fallavier, France) and prepared in dimethyl sulfoxide (DMSO). Anti-Cyclin A antibody (sc-751; 1:1000), Cyclin B (sc-752; 1:1000), Cyclin E (sc-481; 1:1000), Cdk1 (sc-54; 1:1000), Cdk2 (sc-6248; 1:500), and HSC-70 (sc-7298; 1:1000) were obtained from Santa Cruz Biotechnology (Nanterre, France). Anti-PARP antibody (#9542; 1:1000), Caspase 3 (#9662; 1:1000), Caspase 8 (#4790; 1:1000), Caspase 9 (#9502; 1:1000), BCRP (#42078; 1:1000), and MRP2 (#4446; 1:1000) were purchased from Cell Signaling Technology (Ozyme, Saint-Cyr-l’École, France). Anti-MRP1 antibody (ab24102; 1:50) and MRP3 (ab3375; 1:50) were obtained from Abcam (Paris, France). Anti-P-gp antibody (#MA1-26528; 1:1000) was obtained from Invitrogen/Thermo Fisher Scientific (Paris, France).

### 4.3. Extraction of Essential Oils

The essential oils from *Pituranthus Chlorantus* (PC) and *Teucrium ramosissimum* (TR) were extracted and analyzed as previously published [23,62]. Briefly, after collection, PC and TR leaves were dried in the dark at room temperature and have next been reduced to a fine powder from which the essential oils have been extracted. A steam distillation-based extraction was performed for 3 h before drying the oils’ tanks to anhydrous sodium sulphate. PC and TR essential oils were kept at 4 °C in sterile conditions before their composition analysis by Gas Chromatography (GC)-Mass Spectrometry (MS), as described in [23,62]. The compounds representing at least 1% of the area of total volatiles detected are summarized in Supplementary Table S1. For all experiments, essential oils (PC and TR) were prepared in DMSO.

### 4.4. Cell Viability Assays

MES-SA and MES-SA/Dx5, BMDM, and HUF cell lines were seeded in 96-well plates and incubated for 24 h. Cells were then treated with increasing concentrations of PC and TR for 72 h. MES-SA and MES-SA/Dx5 cells were also treated with increasing concentrations of Dox, cisplatin, 5-fluoro-uracil, and paclitaxel for 72 h. Cell viability was determined through the colorimetric methylthiazol tetrazolium (MTT) assay. This method is based on the ability of mitochondrial succinate deshydrogenases in living cells to reduce a tetrazolium salt, 3-(4,5-dimethylthiazol-2-yl)-2,5-diphenyltetrazolium bromide, leading to the production of blue formazan crystals [63]. Thus, this quantitative colorimetric assay has been widely used to assess the effects of drugs on cell growth [64]. After MTT addition (0.5 mg/mL), the plates were incubated at 37 °C in a 5% CO_2_ humidified atmosphere. After a 3 h incubation, the medium was removed, and formazan crystals were dissolved with acidic isopropanol (0.1 N HCl in absolute isopropanol). Absorbance at 570 nm was measured using the Biochrom Asys UVM 340 microplate reader (Biochrom Ltd., Holliston, MA, USA). Results are expressed as a percentage of control values. The inhibitory concentrations at 50% (IC_50_) for each drug, defined as the concentration of drug that inhibits cell proliferation by 50%, were calculated using a four-parameter nonlinear regression with GraphPad Prism version 6 software (GraphPad Software, San Diego, CA, USA). The resistance index (RI) was calculated by the ratio of the IC_50_ of resistant cells to that of parental cells.

### 4.5. Cell Cycle Analysis by Flow Cytometry

Cell cycle was analyzed by flow cytometry by quantitation of DNA content after propidium iodide (PI) dye staining. Briefly, after seeding and overnight recovering, sensitive (MES-SA) and resistant (MES-SA/Dx5) cells were exposed for 72 h to PC and TR extracts at the concentrations of 12.5, 25, and 50 µg/mL. At the end of the treatments, cells were harvested and washed 3 times with ice-cold PBS before fixation/permeabilization with 70% ethanol at −20 °C. After 24 h, cells were washed with PBS and then stained for 1 h at 37 °C with a solution containing 200 μg/mL RNase A and 50 μg/mL PI (Sigma-Aldrich, St. Quentin Fallavier, France). At the end of the incubation, cells were washed with cold PBS, and then PI fluorescence, varying according to DNA content and cell cycle phase, was measured with a BD FACSCanto^TM^ cytometer equipped with BD FACSDiva software v.8.0.3 (BD Biosciences, Le Pont de Claix, France) and analyzed with the v.10 FlowJo software (Tree Star, Ashland, OR, USA).

### 4.6. Western Blot Analysis 

Cell lysates and immunoblots were performed as described previously [21]. Briefly, MES-SA and MES-SA/Dx5 cells were treated with 12.5, 25, and 50 µg/mL of PC and TR for 72 h before protein extraction with radioimmunoprecipitation assay (RIPA) buffer supplemented with a complete phosphatase and protease inhibitor cocktail, (Roche, Boulogne-Billancourt, France). Protein quantification was performed using the QuantiProTM BCA assay kit (Sigma-Aldrich, St. Quentin Fallavier, France). Samples containing 20–60 µg of proteins were prepared in Laemmli gel loading buffer and then heated for 5 min at 95 °C (except for ABC transporter analysis). Proteins were resolved by sodium dodecyl sulfate–polyacrylamide gel electrophoresis (SDS-PAGE) and transferred to nitrocellulose membranes (Amersham, Les Ulis, France). Blots were then saturated with 5% milk for 1 h at room temperature, followed by an incubation with specific primary antibodies. After the overnight incubation at 4 °C, primary antibodies were detected using horseradish peroxidase (HRP)-conjugated secondary antibodies (Jackson ImmunoResearch, Interchim, Montlucon, France) for 1 h at room temperature, before exposure to enhanced chemiluminescence (ECL) (Bio-Rad, Marnes-la-Coquette, France). A signal was acquired with a ChemiDoc^TM^ XRS + imaging system (Bio-Rad, Marnes-la-Coquette, France), and blots were analyzed with Image Lab^TM^ v6.0.1 software (Bio-Rad).

### 4.7. Apoptosis Identification by Fluorescence Microscopy

Apoptosis was assessed by Hoechst 33342 staining following the manufacturer’s protocol (Apoptosis Hoechst staining kit, Beyotime Biotechnology, Jiangsu, China). Briefly, MES-SA and MES-SA/Dx5 cells were seeded in 6-well plates and incubated for 24 h before treatment with 12.5, 25, and 50 µg/mL of PC and TR. After 72 h of treatment, floating and adherent cells were collected, washed twice with PBS, stained with Hoechst 33258 solution (10 µg/mL) for 15 min at room temperature in the dark, and then mounted onto glass slides. Fluorescence was detected using an epi-fluorescence microscope (Zeiss, Oberkochen, Germany). Nuclei presenting chromatin condensation and fragmentation were considered apoptotic.

### 4.8. Apoptosis Analysis by Flow Cytometry

Cell viability was determined using annexin V-FITC/7-amino-actinomycin D (7AAD) staining from BD Biosciences, as previously described [21]. Briefly, MES-SA and MES-SA/Dx5 cells were seeded in 12-well plates. The following day, cells were treated with 12.5, 25, and 50 µg/mL of PC and TR for 72 h. Floating and adherent cells were incubated with 5 µL of annexin V-FITC and 5 µL of 7AAD in 50 µL of binding buffer for 15 min at room temperature in the dark. Thereafter, 200 μL of binding buffer was added and cells were analyzed with a BD FACSCanto^TM^ cytometer (BD Biosciences, Le Pont de Claix, France).

### 4.9. Measurement of Intracellular ROS Accumulation

The intracellular ROS level was measured using a nonfluorescent probe, 2,7-diacetyl dichlorofluorescein (DCFH-DA), which penetrates into the intracellular matrix of cells where it is oxidized by ROS to fluorescent dichlorofluorescein (DCF). MES-SA and MES-SA/Dx5 cells were incubated with 12.5, 25, and 50 µg/mL of PC and TR for 72 h. Thereafter, 5 μL of a 25 μM solution of 2′,7′-dichlorofluorescin diacetate (DCFH-DA; Fluka, Steinheim, Germany) was added to each well. After 1 h of incubation, the fluorescence of each well was followed every 5 min for 1 h using a fluorescence microplate reader (Biotek, Winooski, VT, USA) with 538 nm emission and 485 nm excitation filters. The area under the fluorescence vs. time curve was integrated at each time point to calculate the ROS units. The median effective dose (EC_50_) was determined for the samples tested from the median effect plot of log (fa/fu) vs. log (dose), where fa is the fraction affected and fu is the fraction unaffected by the treatment.

### 4.10. Effect on Mitochondrial Membrane Potential (ΔY_m_)

The effect of PC and TR on mitochondrial membrane potential was measured using rhodamine 123. Briefly, treated cells were harvested, washed twice with PBS, and then incubated at 37 °C for 20 min with staining buffer containing rhodamine 123. Fluorescence intensity was detected using a fluorescence microplate reader (Biotek, Winooski, VT, USA) with 595 nm emission and 488 nm excitation filters.

### 4.11. Combination Index Analysis

MES-SA and MES-SA/Dx5 cells were seeded in 96-well plates and allowed to recover overnight. Cells were then pretreated for 24 h with increasing concentrations of PC or TR, followed by removal of the medium and addition of increasing Dox concentrations for the last 24 and 48 h, before the MTT assay, as described above. The synergism, additivity, and antagonism of the combinations of the two drugs were calculated using CompuSyn v1.0 software (ComboSyn, Inc., Paramus, NJ, USA), as previously described [21], thanks to the determination of combination index (CI) on the basis of the Chou–Talalay method [37,38]. CI values for each fixed-dose combination of the two drugs were plotted against the corresponding effect levels (FA), expressed as a percentage of growth inhibition. The FA-CI values obtained with PC–Dox and TR–Dox combinations (for 48 and 72 h) are summarized in Supplementary Tables S2 and S3, respectively. Drug interactions were also assessed using an isobologram, plotted as of the dose of each drug (D) in combination normalized with the dose (D_x_) of single drugs, at different effect levels (x%), on both the horizontal- and vertical-axes. Moreover, the dose-reduction index (DRI) of each drug can be simulated as the inverted terms of the CI equation, defined as the fold of drug 1 (or 2) dose reduction allowed by the synergistic combination, while maintaining the same % inhibition as single drug 1 (or 2). As with the FA-CI plot and normalized isobologram, the FA-DRI plot was generated using CompuSyn (ComboSyn, Inc., Paramus, NJ, USA).

### 4.12. P-gp ATPase Activity Assay

The P-gp ATPase activity assay was performed using the luminescent P-gp-Glo^TM^ Assay System according to the manufacturer’s instructions (Promega, Charbonnières-les-Bains, France). This assay reveals the capacity of compounds to stimulate or inhibit ATPase activity of recombinant human P-gp in a cell membrane fraction. ATP is first incubated with P-gp, and then the P-gp ATPase reaction is stopped. The remaining unmetabolized ATP is detected as a luciferase-generated luminescent signal. Thus, a P-gp-dependent decrease in luminescence reflects ATP consumption by P-gp, indicating an increase in P-gp activity. The assay includes controls such as an untreated sample, verapamil, and sodium orthovanadate (Na_3_VO_4_). Verapamil is a stimulator of P-gp ATPase activity that will competitively inhibit the efflux of other compounds and is used as a positive control. Na_3_VO_4_ selectively inhibits P-gp ATPase activity (negative control). The test compounds are essential oils (PC and TR) alone and in combination with verapamil. First, the untreated sample, 100 µM Na_3_VO_4_, 200 µM verapamil, and 50 µg/mL PC and TR alone were pre-incubated with P-gp membranes in an untreated white opaque 96-well plate for approximately 5 min at 37 °C. MgATP was then added to initiate the reaction, followed by incubation for 2 h at 37 °C. Concerning the PC/TR-verapamil combination, PC and TR were incubated alone for 1.5 h before the addition of verapamil for 30 min. Subsequently, the P-gp reactions were stopped and luminescence was initiated by addition of ATP detection reagent to all wells. Following a 20 min signal development period at room temperature, luminescence was read on an EnVision^®^ Multimode Plate Reader (PerkinElmer, Villebon-sur-Yvette, France). The difference in the average luminescence intensity between Na_3_VO_4_-treated samples and untreated samples (i.e., ∆RLU_basal_) reflects basal P-gp ATPase activity. The difference in the average luminescent signal between Na_3_VO_4_-treated samples and samples treated with the test compound (TC) (i.e., ∆RLU_TC_) represents drug-related P-gp ATPase activity. The comparison between basal activity and compound-related activity indicates a stimulatory/inhibitory effect or no effect on P-gp ATPase activity. Finally, the fold change in P-gp ATPase activity induced by the test compounds was determined by dividing ∆RLU_TC_ by ∆RLU_basal_.

### 4.13. Doxorubicin Uptake Analysis

MES-SA/Dx5 cells were seeded in 12-well plates and incubated in culture medium containing 12.5, 25, and 50 µg/mL of PC and TR. Following 1 h of incubation, cells were incubated with Dox (5 μM) for 6 h at 37 °C. Thereafter, cells were washed, and the medium was replaced by drug-free culture medium for 1 h at 37 °C. Cells were then harvested and washed twice with PBS followed by the intracellular Dox analysis with a FACS Aria flow cytometer (BD Biosciences, Le Pont de Claix, France) with excitation and emission wavelengths of 470 and 585 nm.

### 4.14. Statistical Analysis

Data are represented as mean ± standard deviation (SD) or standard error of the mean (SEM) of at least three independent experiments. Statistical analyses were performed with GraphPad Prism v6 software (GraphPad Software, San Diego, CA, USA). Continuous data were compared using a one-way or two-way ANOVA as appropriate, followed by Tukey’s multiple comparison test after confirming a normal distribution and variance homogeneity. All *p*-values are two-tailed, and *p*-values less than 0.05 were considered significant (* *p* < 0.05, ** *p* < 0.01, and *** *p* < 0.001).

## 5. Conclusions

Drug resistance accounts for poor treatment outcomes and tumor relapse. These first results highlight the value of Apiaceae extracts, *Pituranthos chloranthus* (*PC*) and *Teucrium ramosissimum* Desf. (*TR*), for enhancing the anticancer activity of doxorubicin in resistant uterine sarcoma MES-SA/Dx5 cells overexpressing P-gp protein. This study underlines the potential use of PC and TR as a chemosensitizer in combination with doxorubicin to decrease the toxicity of the latter over the long term. Therefore, additional studies should be conducted with in vivo assays to demonstrate the potential clinical use of PC or TR in new anticancer strategies.

## Figures and Tables

**Table 1:** The resistance index (RI) for various chemotherapies.

Drug	IC_50_ (µM)		RI *
	MES-SA	MES-SA/Dx5	
Cisplatin	4.12 ± 0.01	5.01 ± 0.02	1.21 ± 0.2
5-Fluorouracil	1.05 ± 0.05	2.5 ± 0.01	2.38 ± 0.2
Paclitaxel	0.09 ± 0.1	0.35 ± 0.08	3.8 ± 0.8
Doxorubicin	0.074 ± 0.02	0.8 ± 0.01	10.8 ± 0.5

* Resistance index (RI): ratio between the IC_50_ of resistant cells and that of the parental cells. IC_50_: Inhibitory concentration 50%.

**Table 2:** The resistance index (RI) for PC and TR after 72 h of treatment.

Drug	IC_50_ (µg/mL)		RI *
	MES-SA	MES-SA/Dx5	
PC	33.50 ± 0.55	57.56 ± 1.87	1.72 ± 0.09
TR	18.64 ± 1.21	47.39 ± 3.85	2.56 ± 0.38

* Resistance index (RI): ratio between the IC_50_ of resistant cells and that of the parental cells. IC_50_: Inhibitory concentration 50%.

## Data Availability

Data is contained within the article and Supplementary Materials.

## Supplementary Materials

The following are available online at https://www.mdpi.com/article/10.3390/nu13051719/s1, Figure S1: Inhibitory effects of Doxorubicin on MES-SA and MES-SA/Dx5 cell proliferation after 48 h of treatment; Figure S2: Effects of PC and TR extracts on cell cycle distribution in MES-SA and MES-SA/Dx5 cell lines; Figure S3: Quantifications of the protein expressions of key cell cycle regulators in MES-SA and MES-SA/Dx5 cells after a 72 h PC and TR treatment; Figure S4: Quantifications of the protein expressions of the key players of apoptosis in MES-SA cells after a 72 h PC and TR treatment; Figure S5: Quantifications of the protein expressions of the key players of apoptosis in MES-SA/Dx5 cells after a 72 h PC and TR treatment; Figure S6: Determination of the combinatory effect of PC or TR with doxorubicin (Dox) on MES-SA/Dx5 cell viability. Table S1: Chemical composition of essential oils (% area of total volatiles) detected from *Pituranthos chloranthus* and *Teucrium ramosissimum*; Table S2: Dose-response relation of the combination PC-Dox against MES-SA/Dx5 cells, after 48 and 72 h of incubation; Table S3: Dose-response relation of the combination TR-Dox against MES-SA/Dx5 cells, after 48 and 72 h of incubation.

## References

[1] Gottesman M.M. (2002). Mechanisms of Cancer Drug Resistance. Annu. Rev. Med..

[2] Giaccone G., Pinedo H.M. (1996). Drug Resistance. Oncology.

[3] Larsen A.K., Escargueil A.E., Skladanowski A. (2000). Resistance mechanisms associated with altered intracellular distribution of anticancer agents. Pharmacol. Ther..

[4] Triller N., Korosec P., Kern I., Kosnik M., Debeljak A. (2006). Multidrug resistance in small cell lung cancer: Expression of P-glycoprotein, multidrug resistance protein 1 and lung resistance protein in chemo-naive patients and in relapsed disease. Lung Cancer.

[5] Bukowski K., Kciuk M., Kontek R. (2020). Mechanisms of Multidrug Resistance in Cancer Chemotherapy. Int. J. Mol. Sci..

[6] Curtin N.J. (2012). DNA repair dysregulation from cancer driver to therapeutic target. Nat. Rev. Cancer.

[7] Hanahan D., Weinberg R.A. (2011). Hallmarks of Cancer: The Next Generation. Cell.

[8] Szakács G., Paterson J.K., Ludwig J.A., Booth-Genthe C., Gottesman M.M. (2006). Targeting multidrug resistance in cancer. Nat. Rev. Drug Discov..

[9] Aires V., Colin D.J., Doreau A., Di Pietro A., Heydel J.-M., Artur Y., Latruffe N., Delmas D. (2019). P-Glycoprotein 1 Affects Chemoactivities of Resveratrol against Human Colorectal Cancer Cells. Nutrients.

[10] Housman G., Byler S., Heerboth S., Lapinska K., Longacre M., Snyder N., Sarkar S. (2014). Drug Resistance in Cancer: An Overview. Cancers.

[11] Zagouri F., Dimopoulos A.-M., Fotiou S., Kouloulias V., Papadimitriou C.A. (2009). Treatment of early uterine sarcomas: Disentangling adjuvant modalities. World J. Surg. Oncol..

[12] Trope C.G., Abeler V.M., Kristensen G.B. (2012). Diagnosis and treatment of sarcoma of the uterus. A review. Acta Oncol..

[13] Kyriazoglou A., Liontos M., Ziogas D.C., Zagouri F., Koutsoukos K., Tsironis G., Tsiara A., Kaparelou M., Zakopoulou R., Thomakos N. (2018). Management of uterine sarcomas and prognostic indicators: Real world data from a single-institution. BMC Cancer.

[14] Hanna A.D., Lam A., Tham S., Dulhunty A.F., Beard N.A. (2014). Adverse Effects of Doxorubicin and Its Metabolic Product on Cardiac RyR2 and SERCA2A. Mol. Pharmacol..

[15] Chatterjee K., Zhang J., Honbo N., Karliner J.S. (2010). Doxorubicin Cardiomyopathy. Cardiology.

[16] Aires V., Limagne E., Cotte A.K., Latruffe N., Ghiringhelli F., Delmas D. (2013). Resveratrol metabolites inhibit human metastatic colon cancer cells progression and synergize with chemotherapeutic drugs to induce cell death. Mol. Nutr. Food Res..

[17] Colin D., Gimazane A., Lizard G., Izard J.-C., Solary E., Latruffe N., Delmas D. (2009). Effects of resveratrol analogs on cell cycle progression, cell cycle associated proteins and 5fluoro-uracil sensitivity in human derived colon cancer cells. Int. J. Cancer.

[18] Colin D.J., Limagne E., Ragot K., Lizard G., Ghiringhelli F., Solary E., Chauffert B., Latruffe N., Delmas D. (2014). The role of reactive oxygen species and subsequent DNA-damage response in the emergence of resistance towards resveratrol in colon cancer models. Cell Death Dis..

[19] Solary E., Latruffe N. (2011). Resveratrol, a Phytochemical Inducer of Multiple Cell Death Pathways: Apoptosis, Autophagy and Mitotic Catastrophe. Curr. Med. Chem..

[20] Delmas D., Xiao J. (2012). Natural Polyphenols Properties: Chemopreventive and Chemosensitizing Activities. Anticancer Agents Med. Chem..

[21] Scagliarini A., Mathey A., Aires V., Delmas D. (2020). Xanthohumol, a Prenylated Flavonoid from Hops, Induces DNA Damages in Colorectal Cancer Cells and Sensitizes SW480 Cells to the SN38 Chemotherapeutic Agent. Cells.

[22] Sioud F., Amor S., Ben Toumia I., Lahmar A., Aires V., Chekir-Ghedira L., Delmas D. (2020). A New Highlight of Ephedra alata Decne Properties as Potential Adjuvant in Combination with Cisplatin to Induce Cell Death of 4T1 Breast Cancer Cells In Vitro and In Vivo. Cells.

[23] Lahmar A., Bedoui A., Mokdad-Bzeouich I., Dhaouifi Z., Kalboussi Z., Cheraif I., Ghedira K., Chekir-Ghedira L. (2017). Reversal of resistance in bacteria underlies synergistic effect of essential oils with conventional antibiotics. Microb. Pathog..

[24] Ben Sghaier M., Chraief I., Skandrani I., Bouhlel I., Boubaker J., Kilani S., Neffati A., Mahmoud A., Hammami M., Chekir-Ghedira L. (2007). Chemical Composition and Antimicrobial Activity of the Essential Oil ofTeucrium ramosissimum (Lamiaceae). Chem. Biodivers..

[25] Ben Sghaier M., Mousslim M., Pagano A., Ammari Y., Luis J., Kovacic H. (2016). β-eudesmol, a sesquiterpene from Teucrium ramosissimum, inhibits superoxide production, proliferation, adhesion and migration of human tumor cell. Environ. Toxicol. Pharmacol..

[26] Shapira S., Pleban S., Kazanov D., Tirosh P., Arber N. (2016). Terpinen-4-ol: A Novel and Promising Therapeutic Agent for Human Gastrointestinal Cancers. PLoS ONE.

[27] Yu X., Lin H., Wang Y., Lv W., Zhang S., Qian Y., Deng X., Feng N., Yu H., Qian B. (2018). D-limonene exhibits antitumor activity by inducing autophagy and apoptosis in lung cancer. OncoTargets Ther..

[28] Wesolowska O., Paprocka M., Kozlak J., Motohashi N., Dus D., Michalak K. (2005). Human sarcoma cell lines MES-SA and MES-SA/Dx5 as a model for multidrug resistance modulators screening. Anticancer. Res..

[29] Harker W.G., Mackintosh F.R., Sikic B.I. (1983). Development and characterization of a human sarcoma cell line, MES-SA, sensitive to multiple drugs. Cancer Res..

[30] Harker W.G., Sikic B.I. (1985). Multidrug (pleiotropic) resistance in doxorubicin-selected variants of the human sarcoma cell line MES-SA. Cancer Res..

[31] Hua J., Mutch D.G., Herzog T.J. (2005). Stable suppression of MDR-1 gene using siRNA expression vector to reverse drug resistance in a human uterine sarcoma cell line. Gynecol. Oncol..

[32] Morgan D.O. (1995). Principles of CDK regulation. Nat. Cell Biol..

[33] Delmas D., Rébé C., Lacour S., Filomenko R., Athias A., Gambert P., Cherkaoui-Malki M., Jannin B., Dubrez-Daloz L., Latruffe N. (2003). Resveratrol-induced Apoptosis Is Associated with Fas Redistribution in the Rafts and the Formation of a Death-inducing Signaling Complex in Colon Cancer Cells. J. Biol. Chem..

[34] Doria M., Nury T., Delmas D., Moreau T., Lizard G., Vejux A. (2019). Protective function of autophagy during VLCFA-induced cytotoxicity in a neurodegenerative cell model. Free. Radic. Biol. Med..

[35] Rastogi R.P., Singh S.P., Häder D.-P., Sinha R.P. (2010). Detection of reactive oxygen species (ROS) by the oxidant-sensing probe 2′,7′-dichlorodihydrofluorescein diacetate in the cyanobacterium Anabaena variabilis PCC 7937. Biochem. Biophys. Res. Commun..

[36] Crompton M. (1999). The mitochondrial permeability transition pore and its role in cell death. Biochem. J..

[37] Chou T.-C. (2010). Drug Combination Studies and Their Synergy Quantification Using the Chou-Talalay Method. Cancer Res..

[38] Chou T.-C. (2006). Theoretical Basis, Experimental Design, and Computerized Simulation of Synergism and Antagonism in Drug Combination Studies. Pharmacol. Rev..

[39] Abeler V.M., Røyne O., Thoresen S., Danielsen H.E., Nesland J.M., Kristensen G.B. (2009). Uterine sarcomas in Norway. A histopathological and prognostic survey of a total population from 1970 to 2000 including 419 patients. Histopathology.

[40] Amant F., Coosemans A., Debiec-Rychter M., Timmerman D., Vergote I. (2009). Clinical management of uterine sarcomas. Lancet Oncol..

[41] Alfarouk K.O., Stock C.-M., Taylor S., Walsh M., Muddathir A.K., Verduzco D., Bashir A.H.H., Mohammed O.Y., ElHassan G.O., Harguindey S. (2015). Resistance to cancer chemotherapy: Failure in drug response from ADME to P-gp. Cancer Cell Int..

[42] Bai Z., Gao M., Zhang H., Guan Q., Xu J., Li Y., Qi H., Li Z., Zuo D., Zhang W. (2017). BZML, a novel colchicine binding site inhibitor, overcomes multidrug resistance in A549/Taxol cells by inhibiting P-gp function and inducing mitotic catastrophe. Cancer Lett..

[43] Fröhlich L.F., Mrakovcic M., Smole C., Lahiri P., Zatloukal K. (2014). Epigenetic Silencing of Apoptosis-Inducing Gene Expression Can Be Efficiently Overcome by Combined SAHA and TRAIL Treatment in Uterine Sarcoma Cells. PLoS ONE.

[44] Lorigan P., Verweij J., Papai Z., Rodenhuis S., Le Cesne A., Leahy M.G., Radford J.A., Van Glabbeke M.M., Kirkpatrick A., Hogendoorn P. (2007). Phase III Trial of Two Investigational Schedules of Ifosfamide Compared with Standard-Dose Doxorubicin in Advanced or Metastatic Soft Tissue Sarcoma: A European Organisation for Research and Treatment of Cancer Soft Tissue and Bone Sarcoma Group Study. J. Clin. Oncol..

[45] Bao L., Haque A., Jackson K., Hazari S., Moroz K., Jetly R., Dash S. (2011). Increased Expression of P-Glycoprotein Is Associated with Doxorubicin Chemoresistance in the Metastatic 4T1 Breast Cancer Model. Am. J. Pathol..

[46] Lee G., Joung J.-Y., Cho J.-H., Son C.-G., Lee N. (2018). Overcoming P-Glycoprotein-Mediated Multidrug Resistance in Colorectal Cancer: Potential Reversal Agents among Herbal Medicines. Evidence-Based Complement. Altern. Med..

[47] Syed S.B., Arya H., Fu I.-H., Yeh T.-K., Periyasamy L., Hsieh H.-P., Coumar M.S. (2017). Targeting P-glycoprotein: Investigation of piperine analogs for overcoming drug resistance in cancer. Sci. Rep..

[48] Angelini A., Di Ilio C., Castellani M.L., Conti P., Cuccurullo F. (2010). Modulation of multidrug resistance p-glycoprotein activity by flavonoids and honokiol in human doxorubicin- resistant sarcoma cells (MES-SA/DX-5): Implications for natural sedatives as chemosensitizing agents in cancer therapy. J. Boil. Regul. Homeost. agents.

[49] Cheung K.K.-Y., Chan J.Y.-W., Fung K.-P. (2013). Antiproliferative effect of pheophorbide a–mediated photodynamic therapy and its synergistic effect with doxorubicin on multiple drug-resistant uterine sarcoma cell MES-SA/Dx5. Drug Chem. Toxicol..

[50] D’Anneo A., Carlisi D., Lauricella M., Puleio R., Martinez R., Di Bella S., Di Marco P., Emanuele S., Di Fiore R., Guercio A. (2013). Parthenolide generates reactive oxygen species and autophagy in MDA-MB231 cells. A soluble parthenolide analogue inhibits tumour growth and metastasis in a xenograft model of breast cancer. Cell Death Dis..

[51] Peng T.-I., Jou M.-J. (2004). Mitochondrial Swelling and Generation of Reactive Oxygen Species Induced by Photoirradiation Are Heterogeneously Distributed. Mitochondrial Pathogenesis.

[52] Wartenberg M., Hoffmann E., Schwindt H., Grünheck F., Petros J., Arnold J.R.S., Hescheler J., Sauer H. (2005). Reactive oxygen species-linked regulation of the multidrug resistance transporter P-glycoprotein in Nox-1 overexpressing prostate tumor spheroids. FEBS Lett..

[53] Hoosain F.G., Choonara Y.E., Tomar L.K., Kumar P., Tyagi C., du Toit L.C., Pillay V. (2015). Bypassing P-Glycoprotein Drug Efflux Mechanisms: Possible Applications in Pharmacoresistant Schizophrenia Therapy. BioMed Res. Int..

[54] Callaghan R., Luk F., Bebawy M. (2014). Inhibition of the Multidrug Resistance P-Glycoprotein: Time for a Change of Strategy?. Drug Metab. Dispos..

[55] Cooper J., Giancotti F.G. (2019). Integrin Signaling in Cancer: Mechanotransduction, Stemness, Epithelial Plasticity, and Therapeutic Resistance. Cancer Cell.

[56] Colin D., Limagne E., Jeanningros S., Jacquel A., Lizard G., Athias A., Gambert P., Hichami A., Latruffe N., Solary E. (2011). Endocytosis of Resveratrol via Lipid Rafts and Activation of Downstream Signaling Pathways in Cancer Cells. Cancer Prev. Res..

[57] Brown V.A., Patel K.R., Viskaduraki M., Crowell J.A., Perloff M., Booth T.D., Vasilinin G., Sen A., Schinas A.M., Piccirilli G. (2010). Repeat Dose Study of the Cancer Chemopreventive Agent Resveratrol in Healthy Volunteers: Safety, Pharmacokinetics, and Effect on the Insulin-like Growth Factor Axis. Cancer Res..

[58] Patel K.R., Andreadi C., Britton R.G., Horner-Glister E., Karmokar A., Sale S., Brown V.A., Brenner D.E., Singh R., Steward W.P. (2013). Sulfate Metabolites Provide an Intracellular Pool for Resveratrol Generation and Induce Autophagy with Senescence. Sci. Transl. Med..

[59] Patel K.R., Brown V.A., Jones D.J.L., Britton R.G., Hemingway D., Miller A.S., West K.P., Booth T.D., Perloff M., Crowell J.A. (2010). Clinical Pharmacology of Resveratrol and Its Metabolites in Colorectal Cancer Patients. Cancer Res..

[60] Mukhtar Y.M., Adu-Frimpong M., Xu X., Yu J. (2018). Biochemical significance of limonene and its metabolites: Future prospects for designing and developing highly potent anticancer drugs. Biosci. Rep..

[61] Plengsuriyakarn T., Karbwang J., Na-Bangchang K. (2014). Anticancer activity using positron emission tomography-computed tomography and pharmacokinetics ofβ-eudesmol in human cholangiocarcinoma xenografted nude mouse model. Clin. Exp. Pharmacol. Physiol..

[62] Lahmar A., Morcuende D., Andrade M.J., Chekir-Ghedira L., Estévez M. (2018). Prolonging shelf life of lamb cutlets packed under high-oxygen modified atmosphere by spraying essential oils from North-African plants. Meat Sci..

[63] Mosmann T. (1983). Rapid colorimetric assay for cellular growth and survival: Application to proliferation and cytotoxicity assays. J. Immunol. Methods.

[64] Marshall N.J., Goodwin C.J., Holt S.J. (1995). A critical assessment of the use of microculture tetrazolium assays to measure cell growth and function. Growth Regul..

