# Supplementary material for: Essential Oils, Pituranthos chloranthus and Teucrium ramosissimum, Chemosensitize Resistant Human Uterine Sarcoma MES-SA/Dx5 Cells to Doxorubicin by Inducing Apoptosis and Targeting P-Glycoprotein
Source: Nutrients. 2021 May 19;13(5):1719. doi: 10.3390/nu13051719 (PMC8160977; doi:10.3390/nu13051719)
Supplement: Supplementary file 1 [file nutrients-13-01719-s001.zip › nutrients-1218079-supplementary.pdf]

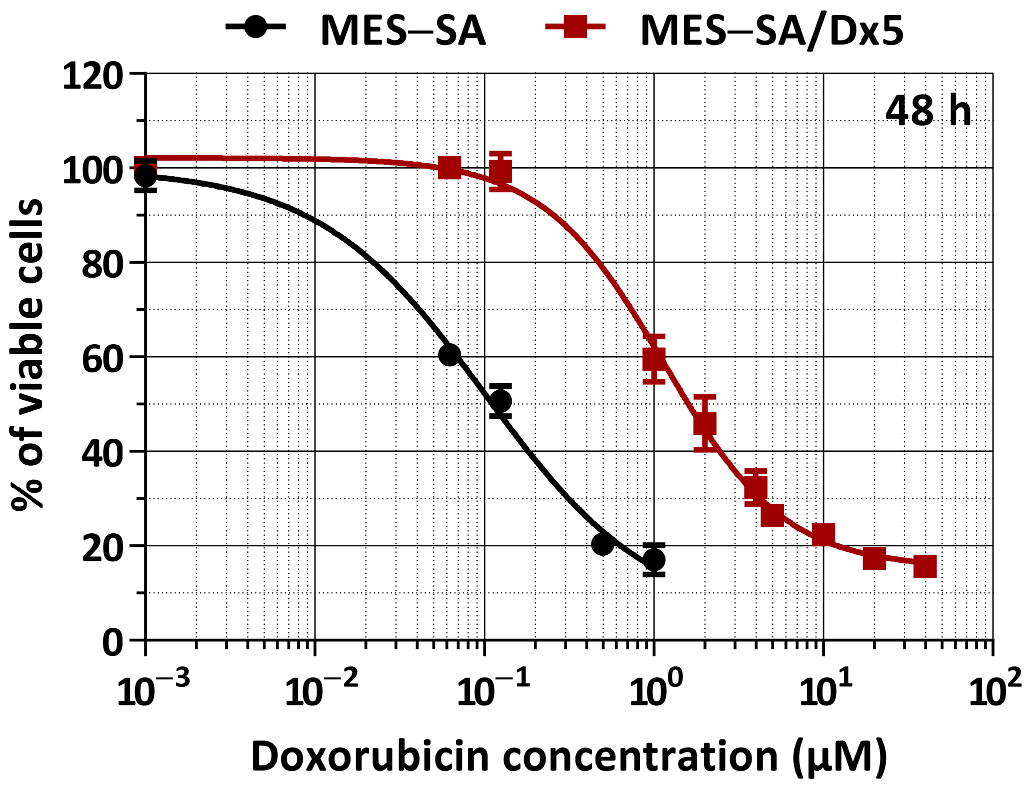

Supplementary Figure S1. Inhibitory effects of Doxorubicin on MES-SA and MES-SA/Dx5 cell proliferation after 48h of treatment. MES-SA and MES-SA/Dx5 were treated with increasing concentrations of doxorubicin for 48h and then, the percentage of cell viability was determined using the (3-(4,5-dimethylthiazol-2-yl)-2,5-diphenyltetrazolium bromide (MTT) assay. The results are expressed as a mean percentage of control growth ± Standard Deviation (SD) of three independent experiments.

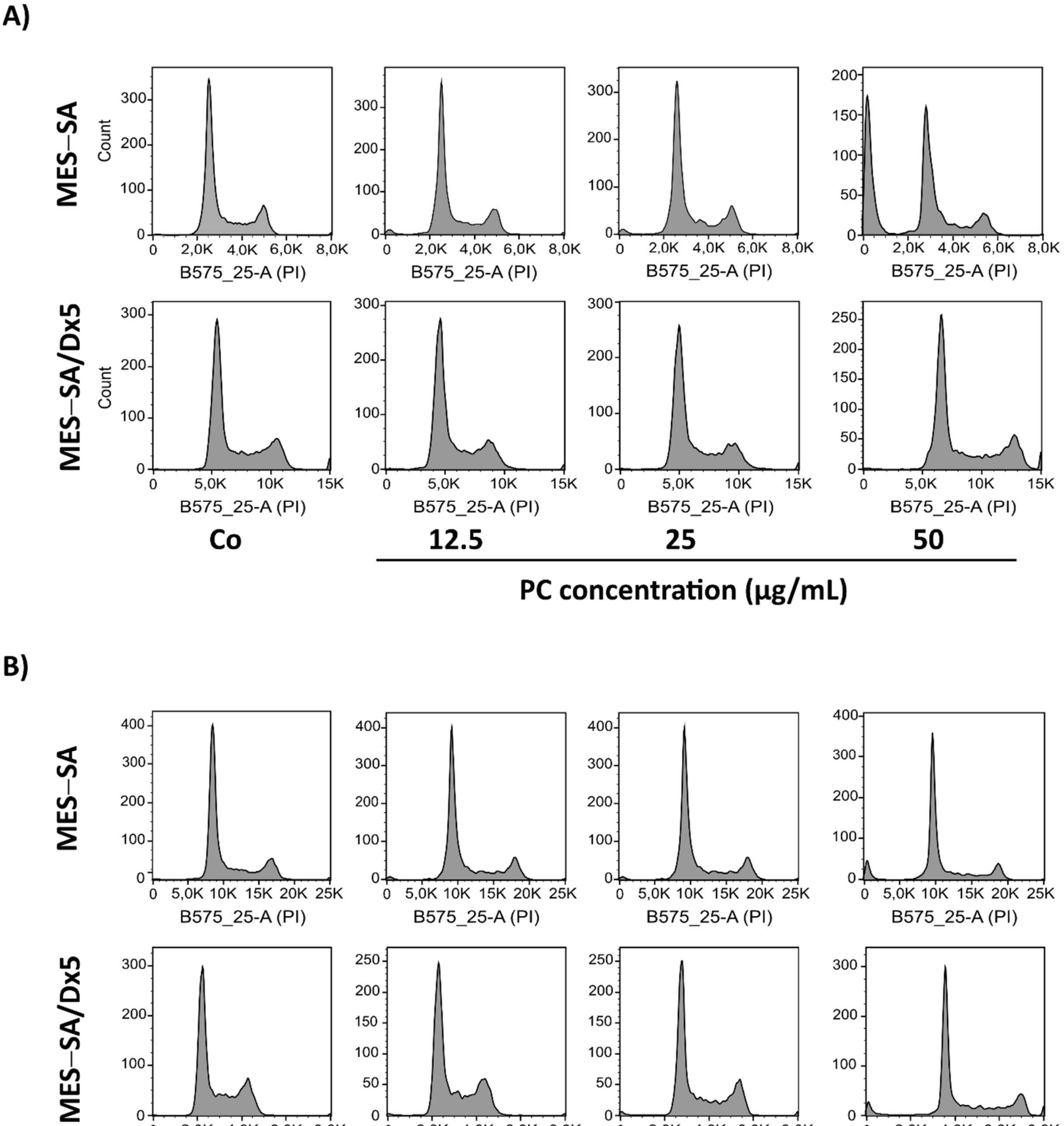

Supplementary Figure S2. Effects of *Pituranthos chloranthus* (PC) and *Teucrium ramosissimum* Desf. (TR) extracts on cell cycle distribution in MES-SA and MES-SA/Dx5 cell lines. After 24 h of culture, uterine sarcoma MES-SA and MES-SA/Dx5 cells were left untreated (0.1% dimethyl sulfoxide [DMSO]; Co, control) or treated with increasing concentrations of PC and TR (12.5, 25, and 50 µg/mL) for 72 h. Cells were thereafter harvested and stained with propidium iodide (PI) and PI signal was next quantified by flow cytometry. (**A**,**B**) Histograms showing MES-SA and MES-SA/Dx5 cell distributions in cell cycle phases after treatment with PC (**A**) and TR (**B**). One representative of three independent experiments is shown.

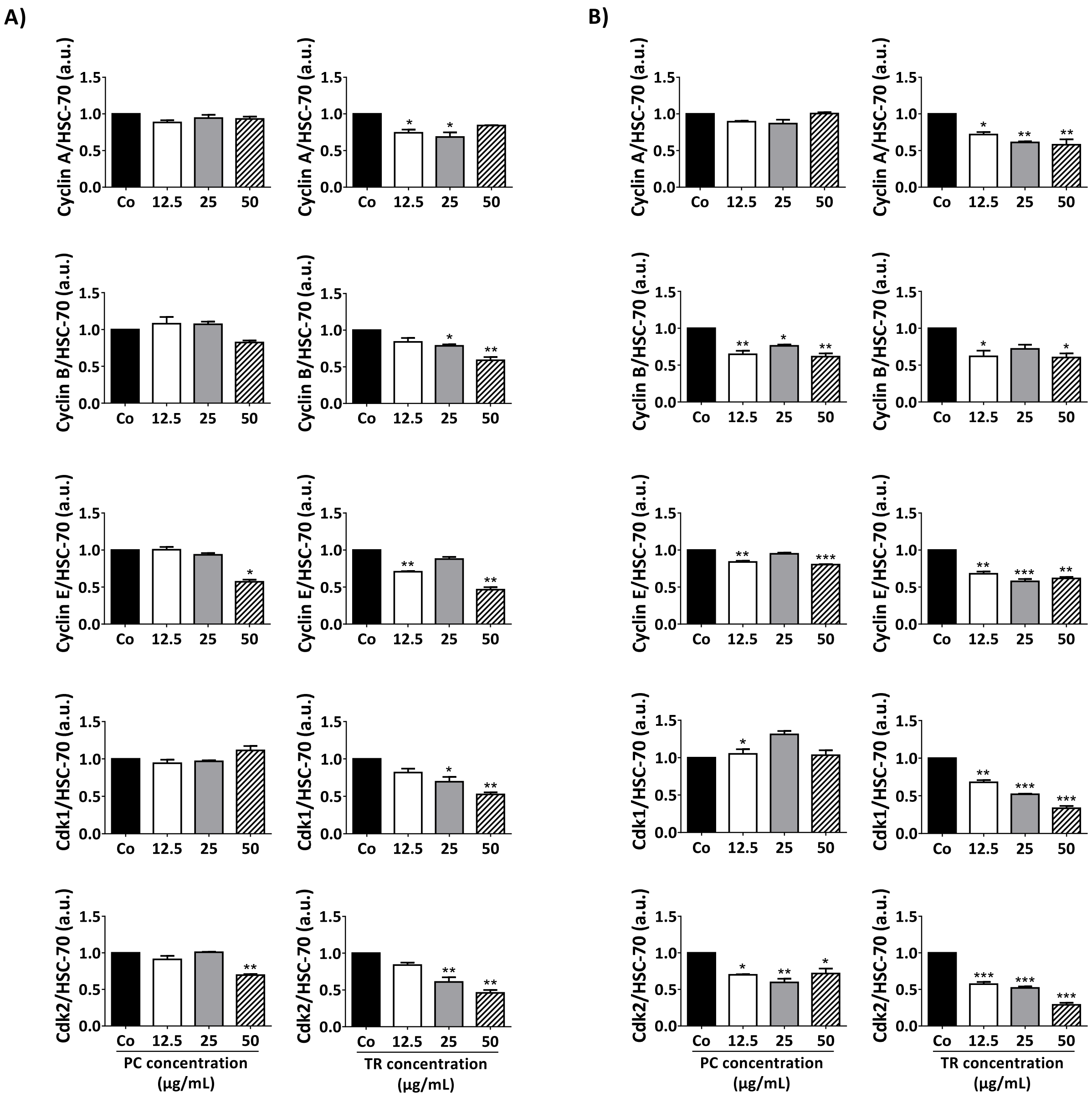

Supplementary Figure S3. Quantifications of the protein expressions of key cell cycle regulators in MES-SA and MES-SA/Dx5 cells after a 72-h *Pituranthos chloranthus* (PC) and *Teucrium ramosissimum* Desf. (TR) treatment. (**A**,**B**) Densitometric quantification of Western blotting obtained, respectively, in (**A**, Figure 5, MES-SA) and (**B**, Figure 5, MES-SA/Dx5). Data are expressed as mean fold induction ± Standard Error of the Mean (SEM) of three independent experiments; p values were determined by a one-way ANOVA followed by Tukey's multiple comparison test. *$p < 0.05$, **$p < 0.01$, and ***$p < 0.001$ compared to control (Co) condition. F values of ANOVA tests for panel A (significant results): Cyclin A expression, F = 12.69 for TR; Cyclin B expression, F = 22.77 for TR; Cyclin E expression, F = 62.35 and F = 103.3 for PC and TR respectively; Cyclin-dependent kinase 1 (Cdk1) expression, F = 20.83 for TR; Cyclin-dependent kinase 2 Cdk2 expression, F = 35.41 and F = 34.31 for PC and TR respectively. F values of ANOVA tests for panel B (significant results): Cyclin A expression, F = 21.79 for TR; Cyclin B expression, F = 26.53 and F = 11.17 for PC and TR respectively; Cyclin E expression, F = 66.33 and F = 69.02 for PC and TR respectively; Cdk1 expression, F = 8.119 and F = 168.8 for PC and TR respectively; Cdk2 expression, F = 16.51 and F = 164.3 for PC and TR respectively.

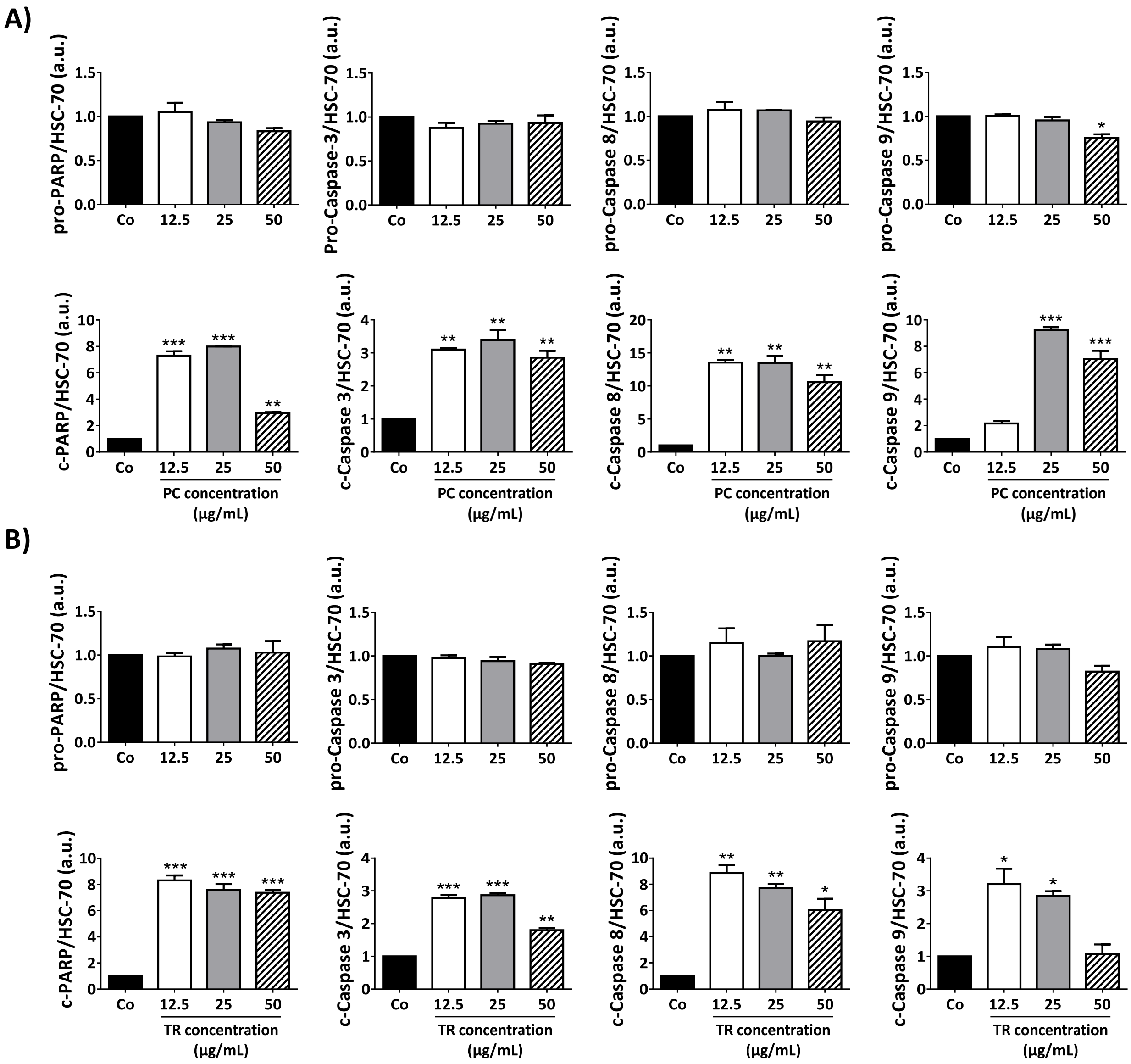

Supplementary Figure S4. Quantifications of the protein expressions of the key players of apoptosis in MES-SA cells after a 72-h *Pituranthos chloranthus* (PC) and *Teucrium ramosissimum* Desf. (TR) treatment. (**A**,**B**) Densitometric quantification of Western blotting obtained in Figure 7A. Data are expressed as mean fold induction ± Standard Error of the Mean (SEM) of three independent experiments; p values were determined using a one-way ANOVA followed by Tukey's multiple comparison test. *$p < 0.05$, **$p < 0.01$, and ***$p < 0.001$ compared to control (Co) condition. F values of ANOVA tests for panel A (PC, significant results): c-Poly(ADP-ribose)-polymerase (c-PARP) expression, F = 421.9; pro-Caspase 9 expression, F = 15.42; c-Caspase 3 expression, F = 34.42; c-Caspase 9 expression, F = 130.6; c-Caspase 8 expression, F = 57.83. F values of ANOVA tests for panel B (TR, significant results): c-PARP expression, F = 120.7; c-Caspase 3 expression, F = 179.1; c-Caspase 9 expression, F = 16.35; c-Caspase 8 expression, F = 38.95.

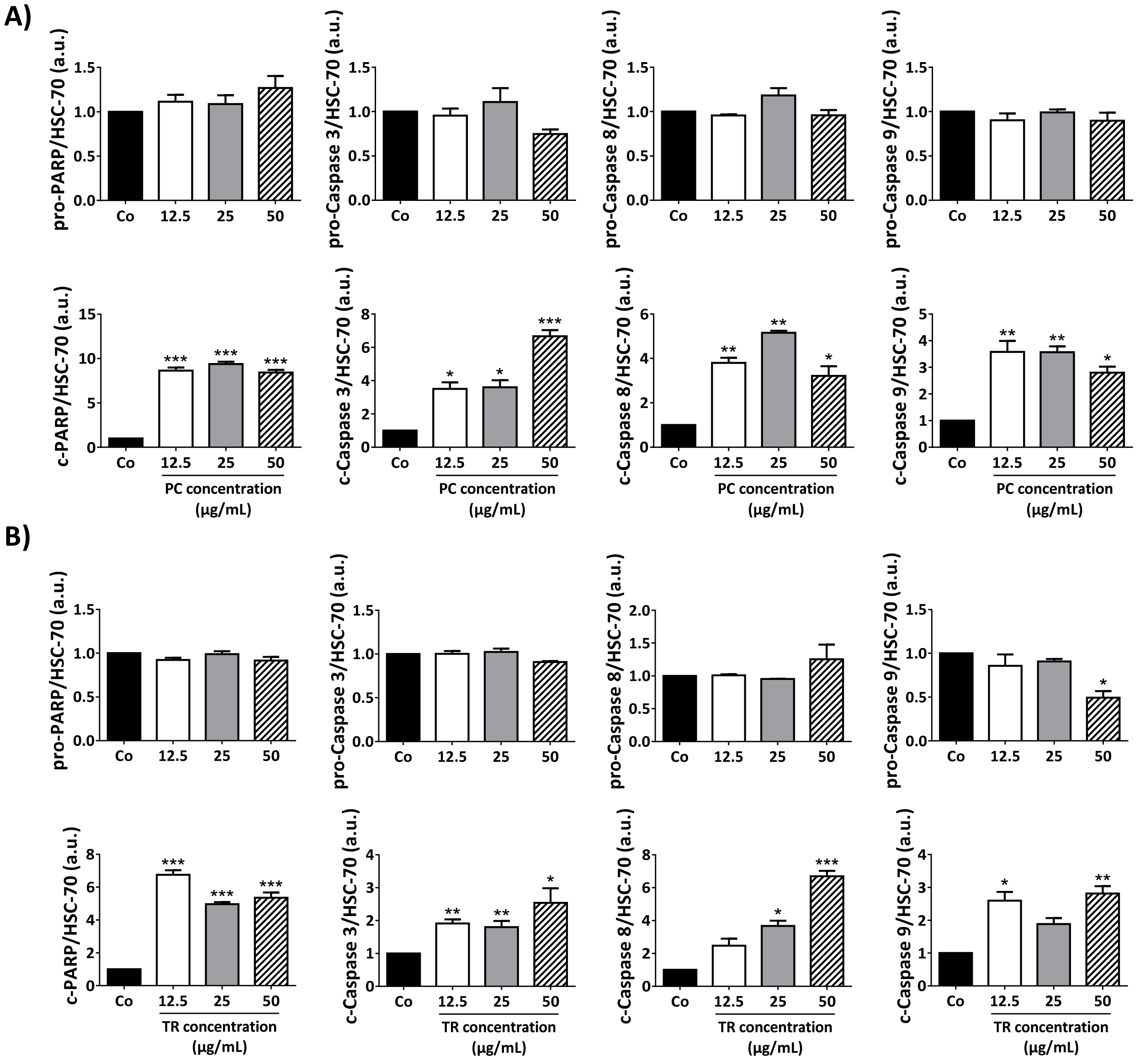

Supplementary Figure S5. Quantifications of the protein expressions of the key players of apoptosis in MES-SA/Dx5 cells after a 72-h *Pituranthos chloranthus* (PC) and *Teucrium ramosissimum* Desf. (TR) treatment. (**A**,**B**) Densitometric quantification of Western blotting obtained, respectively, in Figure 7B. Data are expressed as mean fold induction ± Standard Error of the Mean (SEM) of three independent experiments; p values were determined using a one-way ANOVA followed by Tukey's multiple comparison test. *$p < 0.05$, **$p < 0.01$, and ***$p < 0.001$ compared to control (Co) condition. F values of ANOVA tests for panel A (PC, significant results): c-Poly(ADP-ribose)-polymerase (c-PARP) expression, F = 228.8; c-Caspase 3 expression, F = 46.6; c-Caspase 9 expression, F = 22.02; c-Caspase 8 expression, F = 48.84. F values of ANOVA tests for panel B (TR, significant results): c-PARP expression, F = 123.2; Pro-Caspase 9 expression, F = 8.48; c-Caspase 3 expression, F = 26.36; c-Caspase 9 expression, F = 17.25; c-Caspase 8 expression, F = 59.54.

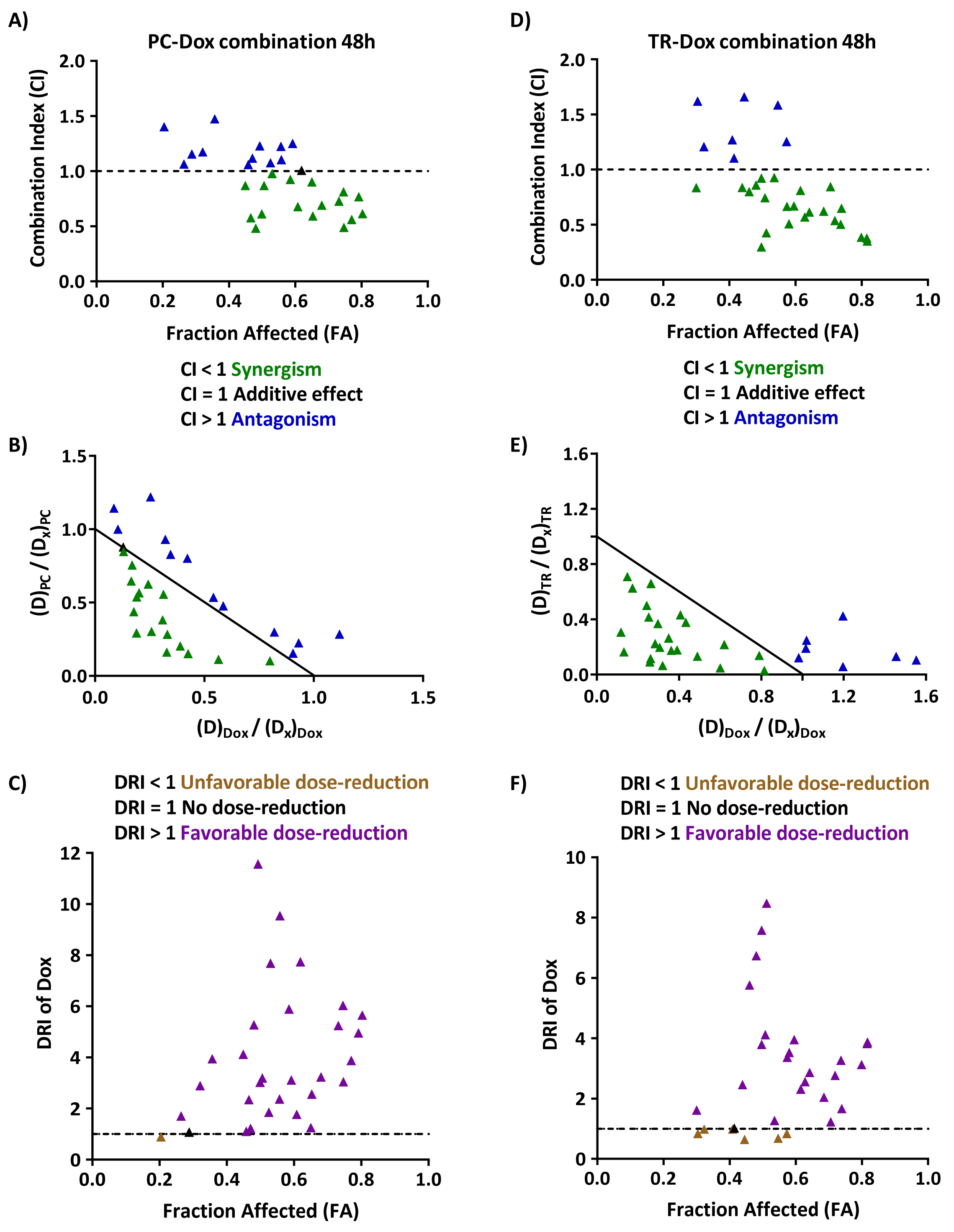

Supplementary Figure S6. Determination of the combinatory effect of of *Pituranthos chloranthus* (PC) and *Teucrium ramosissimum* Desf. (TR) extracts with doxorubicin (Dox) on MES-SA/Dx5 cell viability. Multidrug-resistant MES-SA/Dx5 cells were pretreated with increasing PC or TR concentrations for 24 h. Thereafter, the cells were treated with increasing Dox amounts for the last 24 h. Growth inhibition level of MES-SA/Dx5 cells was determined using the (3-(4,5-dimethylthiazol-2-yl)-2,5-diphenyltetrazolium bromide (MTT) assay. The cytotoxicity curves of single drugs and in combination were then simulated using CompuSyn software. (**A**) Fraction affected (FA) combination index (CI) plot for a 48 h PC–Dox combination. (**B**) Representative normalized isobologram plotted as the dose of each drug (D; PC and Dox) in combination inducing x% inhibition normalized with the dose at x% inhibition (D_x) of single drugs on both the horizontal- and vertical-axes. (**C**) FA-dose reduction index (DRI) plot of Dox at different effect levels (for Dox/PC combinations). (**D**) Fraction affected (FA) combination index (CI) plot

for a 48 h TR–Dox combination. (**E**) Representative normalized isobologram plotted as the dose of each drug (D; TR and Dox) in combination inducing x% inhibition normalized with the dose at x% inhibition (D_x) of single drugs on both the horizontal- and vertical-axes. (**F**) FA-dose reduction index (DRI) plot of Dox at different effect levels (for Dox/TR combinations). DRI = 1 indicates no dose reduction, whereas DRI > 1 and <1 indicate respectively favorable and unfavorable dose reduction. The data of three independent experiments are shown.

Supplementary Table S1. Chemical composition of essential oils (% area of total volatiles) detected from *Pituranthos chloranthus* and *Teucrium ramosissimum*.

N	Compound	Retention time (minutes)	% Area
	Pituranthus chlorantus (PC)		
**1**	Sabinene	5.10	29.86
**2**	Limonene	7.80	16.65
**3**	Cis-Allo-ocimene	10.41	1.77
**4**	Cis-Sabinen hydrate	12.18	3.17
**5**	p-Menth-2- en-1-ol	14.13	2.76
**6**	Terpinen-4-ol	15.66	15.55
**7**	β -Fenchyl Alcohol	16.95	2.22
**8**	Multifidene	18.41	2.03
**9**	Cuminol	19.54	1.60
**10**	Isobenzofuranone	30.61	2.53
**11**	3-Butylidene phthalide	31.58	3.63
	Teucrium ramosissimum (TR)		
**1**	α-Thujene	4.24	7.33
**2**	Sabinene	5.95	7.35
**3**	p-Cymene	8.78	13.05
**4**	Trans-2-caren-4-ol	11.46	1.92
**5**	3-Picoline N-oxide	13.13	1.43
**6**	β-Caryop hyllene	14.64	3.2
**7**	3-Methyl-2-buten-1-ol	16.12	1.33
**8**	γ-Cadinene	20.05	3.65
**9**	1,6-Germacradien-5-ol	23.49	9.97
**10**	α-Selinene	24.02	2.09
**11**	α-Cadinol	27.29	10.74
**12**	β-Eudesmol	28.27	22.14
**13**	Alloaromadendrene	28.92	4.85
**14**	Benzoic acid	30.77	1.50

Only compounds representing at least 1% of the area of total volatiles detected are shown.

Supplementary Table S2. Dose-response relation of the combination PC-Dox against MES-SA/Dx5 cells, after 48 and 72 h of incubation.

Agent		48h		72h	
PC (μg/mL)	Dox (μM)	FA	CI	FA	CI
100	4	0.792	0.768	0.87	0.99
	2	0.746	0.813	0.856	0.584
	1	0.592	1.251	0.766	0.623
	0.5	0.619	1.007	0.728	0.427
	0.25	0.558	1.104	0.711	0.27
	0.125	0.493	1.229	0.651	0.231
80	4	0.803	0.614	0.867	1.017
	2	0.732	0.728	0.822	0.784
	1	0.557	1.224	0.793	0.508
	0.5	0.585	0.925	0.68	0.545
	0.25	0.53	0.977	0.627	0.4
	0.125	0.357	1.474	0.591	0.286
50	4	0.77	0.56	0.882	0.856
	2	0.68	0.691	0.82	0.786
	1	0.525	1.076	0.764	0.606
	0.5	0.506	0.869	0.712	0.435
	0.25	0.449	0.869	0.618	0.387
	0.125	0.321	1.173	0.548	0.308

25	4	0.747	0.49	0.851	1.176
	2	0.653	0.593	0.837	0.673
	1	0.471	1.118	0.702	0.869
	0.5	0.499	0.613	0.713	0.416
	0.25	0.481	0.483	0.531	0.553
	0.125	0.264	1.064	0.525	0.304
12.5	4	0.65	0.902	0.773	2.189
	2	0.608	0.677	0.759	1.206
	1	0.458	1.059	0.693	0.906
	0.5	0.466	0.577	0.642	0.604
	0.25	0.288	1.156	0.409	0.966
	0.125	0.204	1.402	0.259	1.129

FA: Fraction Affected; CI: Combination Index.

Supplementary Table S3. Dose-response relation of the combination TR-Dox against MES-SA/Dx5 cells, after 48 and 72 h of incubation.

**Agent**		**48h**		**72h**	
TR (μg/mL)	Dox (μM)	FA	CI	FA	CI
100	4	0.816	0.377	0.941	0.417
	2	0.737	0.503	0.892	0.57
	1	0.615	0.812	0.838	0.666
	0.5	0.595	0.67	0.835	0.57
	0.25	0.497	0.923	0.827	0.535
	0.125	0.481	0.859	0.759	0.653
80	4	0.817	0.35	0.941	0.368
	2	0.719	0.537	0.878	0.568
	1	0.642	0.614	0.838	0.575
	0.5	0.574	0.665	0.819	0.52
	0.25	0.508	0.744	0.843	0.408
	0.125	0.46	0.8	0.785	0.485
50	4	0.799	0.386	0.944	0.278
	2	0.685	0.622	0.907	0.333
	1	0.628	0.569	0.791	0.597
	0.5	0.58	0.508	0.736	0.571
	0.25	0.439	0.838	0.735	0.448
	0.125	0.512	0.426	0.66	0.531
25	4	0.739	0.649	0.915	0.375
	2	0.547	1.586	0.852	0.468
	1	0.536	0.927	0.726	0.703
	0.5	0.409	1.269	0.717	0.456
	0.25	0.304	1.621	0.549	0.655
	0.125	0.497	0.297	0.453	0.659
12.5	4	0.706	0.844	0.896	0.452
	2	0.573	1.255	0.861	0.377
	1	0.445	1.658	0.735	0.585
	0.5	0.414	1.103	0.714	0.378
	0.25	0.323	1.207	0.482	0.734
	0.125	0.3	0.836	0.247	1.535

FA: Fraction Affected; CI: Combination Index.
